# Low oxygen: A (tough) way of life for Okavango fishes

**DOI:** 10.1371/journal.pone.0235667

**Published:** 2020-07-30

**Authors:** Thea M. Edwards, Ineelo J. Mosie, Brandon C. Moore, Guy Lobjoit, Kelsie Schiavone, Robert E. Bachman, Mike Murray-Hudson

**Affiliations:** 1 Department of Biology, University of the South, Sewanee, Tennessee, United States of America; 2 Okavango Research Institute, University of Botswana, Maun, Botswana; 3 Guma Lagoon Camp, Etsha, Botswana; 4 Department of Chemistry, University of the South, Sewanee, Tennessee, United States of America; Tanzania Fisheries Research Institute, UNITED REPUBLIC OF TANZANIA

## Abstract

Botswana’s Okavango Delta is a World Heritage Site and biodiverse wilderness. In 2016–2018, following arrival of the annual flood of rainwater from Angola’s highlands, and using continuous oxygen logging, we documented profound aquatic hypoxia that persisted for 3.5 to 5 months in the river channel. Within these periods, dissolved oxygen rarely exceeded 3 mg/L and dropped below 0.5 mg/L for up to two weeks at a time. Although these dissolved oxygen levels are low enough to qualify parts of the Delta as a dead zone, the region is a biodiversity hotspot, raising the question of how fish survive. In association with the hypoxia, histological samples, collected from native *Oreochromis andersonii* (threespot tilapia), *Coptodon rendalli* (redbreast tilapia), and *Oreochromis macrochir* (greenhead tilapia), exhibited widespread hepatic and splenic inflammation with marked granulocyte infiltration, melanomacrophage aggregates, and ceroid and hemosiderin accumulations. It is likely that direct tissue hypoxia and polycythemia-related iron deposition caused this pathology. We propose that Okavango cichlids respond to extended natural hypoxia by increasing erythrocyte production, but with significant health costs. Our findings highlight seasonal hypoxia as an important recurring stressor, which may limit fishery resilience in the Okavango as concurrent human impacts rise. Moreover, they illustrate how fish might respond to hypoxia elsewhere in the world, where dead zones are becoming more common.

## Introduction

In northwest Botswana, the Okavango River forms a vast inland oasis as it flows through the arid Kalahari. It is one of the world’s largest wetlands, covering 15,000 to 22,000 square kilometers, depending on season and rainfall. The Okavango is renowned for crystal-clear waters, unhindered and dynamic hydrology, and immense biodiversity, including large numbers of iconic African megafauna [1]. The Ramsar Convention on Wetlands recognizes the Okavango as a *Wetland of International Importance* and, in 2014, the United Nations Educational, Scientific and Cultural Organization (UNESCO) designated the Delta as the 1000th *World Heritage Site*.

**Fig 1:**
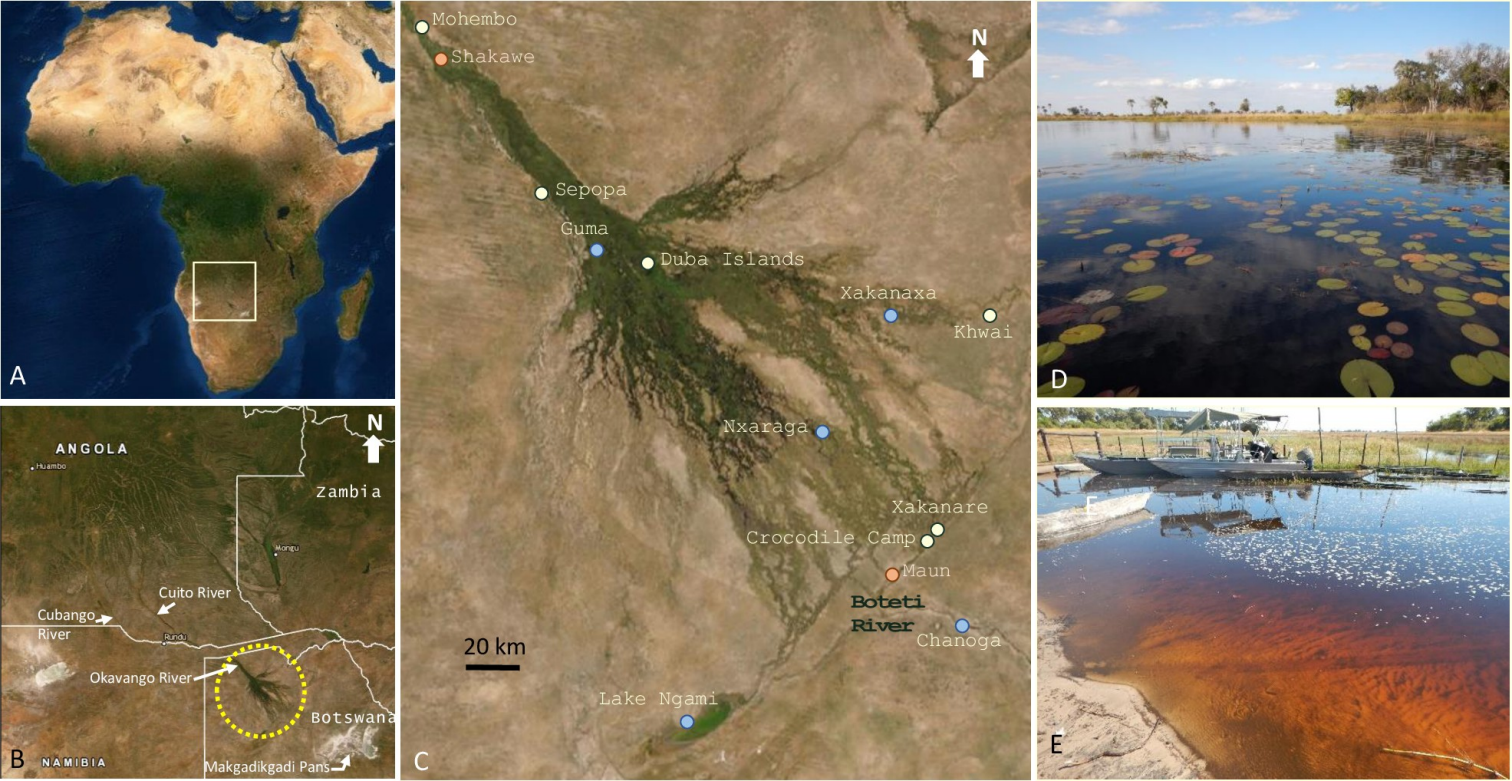
Geography and character of the Okavango Delta. Map of Africa (A) with rectangle that is enlarged in (B) to show the Okavango watershed with major rivers in Angola, Namibia, and Botswana; the yellow dotted circle indicates the Okavango Delta and the bent white arrow shows the Makgadikgadi Pans. Field sites within the Okavango Delta (C) include fish sampling sites shown in blue, major towns in orange, and water quality monitoring sites in yellow and blue; oxygen dataloggers were located at Guma). The Okavango is a vast wetland characterized by exceptional water quality and biodiversity (D). Annual flood arrival is marked by a dramatic increase in dissolved tannins that change the water from clear to tea-colored (E). Photo Credits: TME. Open source map base: https://landlook.usgs.gov/.

An annual cycle of flooding and drying supports Okavango biodiversity. Delta flooding begins in November or December as rainwater drains from the forested mountains of Angola. In Angola, numerous tributaries merge into the Cubango and Cuito Rivers, which merge and flow across the Zambezi Region of Namibia to Botswana, where the river becomes the Okavango (Fig 1A–1C). After entering Botswana, the river flows through the permanent channels and adjacent flood plains of the Delta Panhandle for 90 km between Mohembo and Guma, and then spreads out to fill the alluvial fan of the Delta proper. Because the alluvial fan is almost flat, dropping just 60 meters over its length, the flood water moves slowly, taking six months to travel a linear distance of 256 km, from the Namibia-Botswana border to the town of Maun at the southern end of the Delta [1]. Past Maun, the river continues to Lake Ngami, the Boteti River, and, in a year with plenty of rain, on to the Makgadikgadi Pans (Fig 1B and 1C). The flood peaks in winter (June to August) creating a vast interconnected wetland of channels, islands, lagoons, floating papyrus, and flooded grasslands that is navigable only by small boats and mokoros (canoes) (Fig 1D and 1E).

When the Delta floods, the inundation releases large amounts of dissolved organic matter (DOM) from the floodplains, which have desiccated during the months-long dry season [2]. The hydrated DOM stimulates composting microbes which, in turn, consume most of the available dissolved oxygen (DO) and create a broad band of hypoxia at the leading edge of the flood [1, 2]. This hypoxic band rolls slowly through the Delta such that fishes experience both hypoxic and normoxic “seasons”. In addition to the effects of DOM-fueled respiration, the floating mats of *Cyperus papyrus* and *Phragmites mauririanus* that dominate the channel margins [3] promote hypoxia by slowing water flow and trapping organic matter that decomposes rapidly below water; meanwhile, the bulk of photosynthesis occurs above the water line [4]. All these features conspire to minimize oxygen availability in the water column during the flood season.

During a survey of the Delta Panhandle in March 2016, we encountered this hypoxic band, which occurred throughout the water column in the river channel, and extended for a linear distance of at least 125 km, from Shakawe to Sepopa to Guma, and continuously downstream from Guma to the Duba Islands, including side channels (Fig 1C). We ended our sampling at Duba, but the hypoxia likely continued for some distance. Furthermore, in pockets along the edges of Guma Lagoon, but not elsewhere, hundreds of dead adult tigerfish (*Hydrocynus vittatus*, a top predator) littered the water’s surface. Despite the hypoxia, no other species were found dead in the lagoon or beyond. On the contrary, we were able to catch tilapia (*Oreochromis* and *Coptodon sp*.) in the hypoxic zone.

Hypoxia is an ecologically relevant feature of many aquatic systems in Africa. Among the best studied are the papyrus (*Cyperus sp*.) swamps of East Africa, which exhibit sustained hypoxia with average DO levels below 1.5 mg/L for multi-year periods in some areas [4, 5]. These low oxygen zones serve as refugia that protect hypoxia-adapted fishes from their less tolerant predators, and so play a critical role in the maintenance of biological diversity in these systems [5, 6]. Fishes acclimate to hypoxia through several phenotypically plastic traits, including increased hemoglobin concentration and hematocrit, enlarged gill surface area, reduced critical oxygen tension, and small body size [5], along with adaptive behaviors such as aquatic surface respiration and buccal-bubble holding (gulping air at the water’s surface and holding the bubble in the mouth to enhance oxygenation of the gills) [6]. Fish genera, including *Oreochromis* and *Coptodon*, which fall under the common name of “tilapia” are known to be tolerant to hypoxia and are common native residents of hypoxic African systems [7, 8].

While hypoxia is not universally negative for aquatic species [4], it does induce costs in many organisms, particularly if onset is rapid and animals are not acclimated. In aquaculture and natural systems, hypoxia can rapidly cause mortality, disease susceptibility, changes in gene expression and enzyme activities, and DNA damage, as well as reduce food intake rates–an effect that can cause lower body condition [9–12]. Hypoxia also has far-reaching physiological effects including suppressed reproductive hormones, reduced gamete production, and decreased hepatic glycogen along with increased plasma lactate, glucose, and cortisol; these latter effects derive from induction of anerobic respiration pathways and the stress response [12]. In general, most freshwater organisms require about 5.5 mg/L DO for stress-free living, and the acute mortality limit is considered to be 3 mg/L [13].

Although hypoxia clearly impacts physiology and performance in fishes and is also a feature of the annual Okavango flood, the extent and duration of hypoxic events and impacts on native fish health have never been fully characterized in the Delta. This may be, in part, due to the remoteness of the region (1 to 2 days of backcountry transport to reach most sites) and the lack of backcountry infrastructure (e.g. refrigeration, limited laboratory capabilities), which make physiological studies (e.g. hormones, enzyme activities, gene expression, etc.) logistically challenging. Therefore, to assess fish health, we employed a histological approach because samples could be fixed, stored, and transported at ambient temperatures. Additionally, to assess the duration of flood-related hypoxia, we deployed continuous oxygen loggers in the main channel of the Okavango River at Guma Lagoon (Fig 1C).

## Materials and methods

### Site selection and research permits

Five sites for fish collections were selected along the two main river channels in the Okavango Delta (Fig 1C); they include Guma (GPS coordinates: -18.961702, 22.383521), Xakanaxa (GPS coordinates: -19.189430, 23.404278), Nxaraga (GPS coordinates: -19.548989, 23.176863), Chanoga (GPS coordinates: -20.166765, 23.657201), and Lake Ngami (GPS coordinates: -20.504100, 22.734316). Sampling dates and additional site details are provided in S1 Table. Procedures used for capturing and euthanizing fish for this study followed ethics protocols at the University of Botswana Okavango Research Institute and the University of the South. Fish were euthanized at capture by cervical dislocation. The field research permit (EWT 8/36/4 XXXIII(23)) was approved by Botswana's Ministry of Environment, Wildlife, and Tourism, Private Bag BO 199, Gaborone, Botswana.

### Water quality monitoring

From 2016–2018, DO, conductivity, and water temperatures were monitored at continuous 15 to 20-minute intervals using miniDOT oxygen loggers (Precision Measurement Engineering, Vista, CA) and HOBO U24 freshwater conductivity loggers (Onset, Bourne, MA) with associated company software. The loggers were placed at two sites near Guma camp. At all fish sampling sites, field conductivity, temperature, and pH were measured with a hand-held PCS Testr 35 (Oakton, Vernon Hills, IL). Field DO and temperature were measured with a hand-held YSI model #55 meter (Yellow Springs, OH). All instruments were routinely calibrated as recommended by the manufacturers. Nitrate and nitrite were assessed using Hach Nitrate and Nitrite Test Strips (Cat. 27454–25; Loveland, CO). All conductivity data were corrected to 25°C and presented as specific conductance.

### Fish and tissue sample collections

Adults of three species, *Oreochromis andersonii* (threespot tilapia) (n = 162), *Coptodon rendalli* (redbreast tilapia) (n = 29), and *Oreochromis macrochir* (greenhead tilapia) (n = 32) were collected using 10 cm gill nets placed in shallow channels at each of the five fish sampling sites in the Okavango Delta (Fig 1). Sampling was repeated twice at each site during the flood season (January—July 2016). Nets were set for two to 14 hours (overnight), so not all fish were alive when removed from the net. Fish were euthanized by cervical dislocation, weighed, and measured to obtain total length (TL) (snout to tail tip) and standard length (SL) (snout to caudal peduncle).

Fish were dissected in the field to confirm sex, determine gonad width and reproductive stage (1–5, with 1 being early gamete formation and 5 indicating that a fish was close to spawning), and obtain fresh liver, spleen, and gonad weights. For each animal, gonad widths were measured with calipers at the widest part and averaged to corroborate field gonad staging. Hepatosomatic (HSI), spleen somatic (SSI), and gonadosomatic (GSI) indices were calculated as the ratio of organ weight (mg) to body weight (g), multiplied by 100. Organ biopsies were fixed in 10% formalin and subsequently processed for standard paraffin histology. Additional liver samples and dorso-lateral muscle samples (3–10 g) were either oven-dried at 60°C (Lake Ngami samples only) or salted with non-iodized table salt and ambient-air-dried (all other samples) for later x-ray florescence elemental analysis for heavy metals.

### Histology and pathological scoring

**Fig 2:**
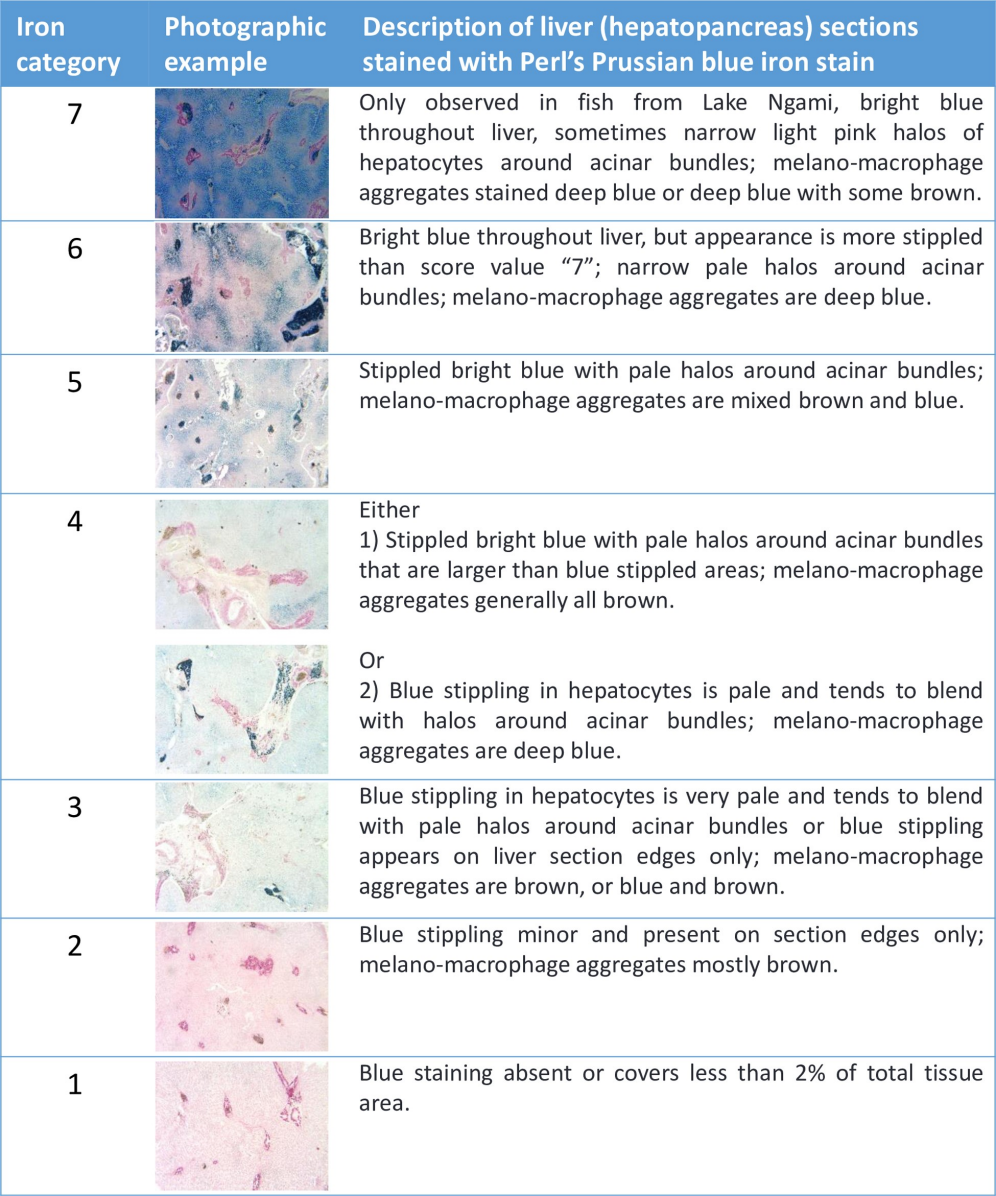
Iron category definitions for liver (hepatopancreas) sections stained with Perl’s Prussian blue iron stain. Categories 1–7 reflect the degree of iron staining in liver sections, with 7 representing the most iron. Category 7 was only observed in Lake Ngami fish. Higher resolution images are available in S1 Fig.

**Fig 3:**
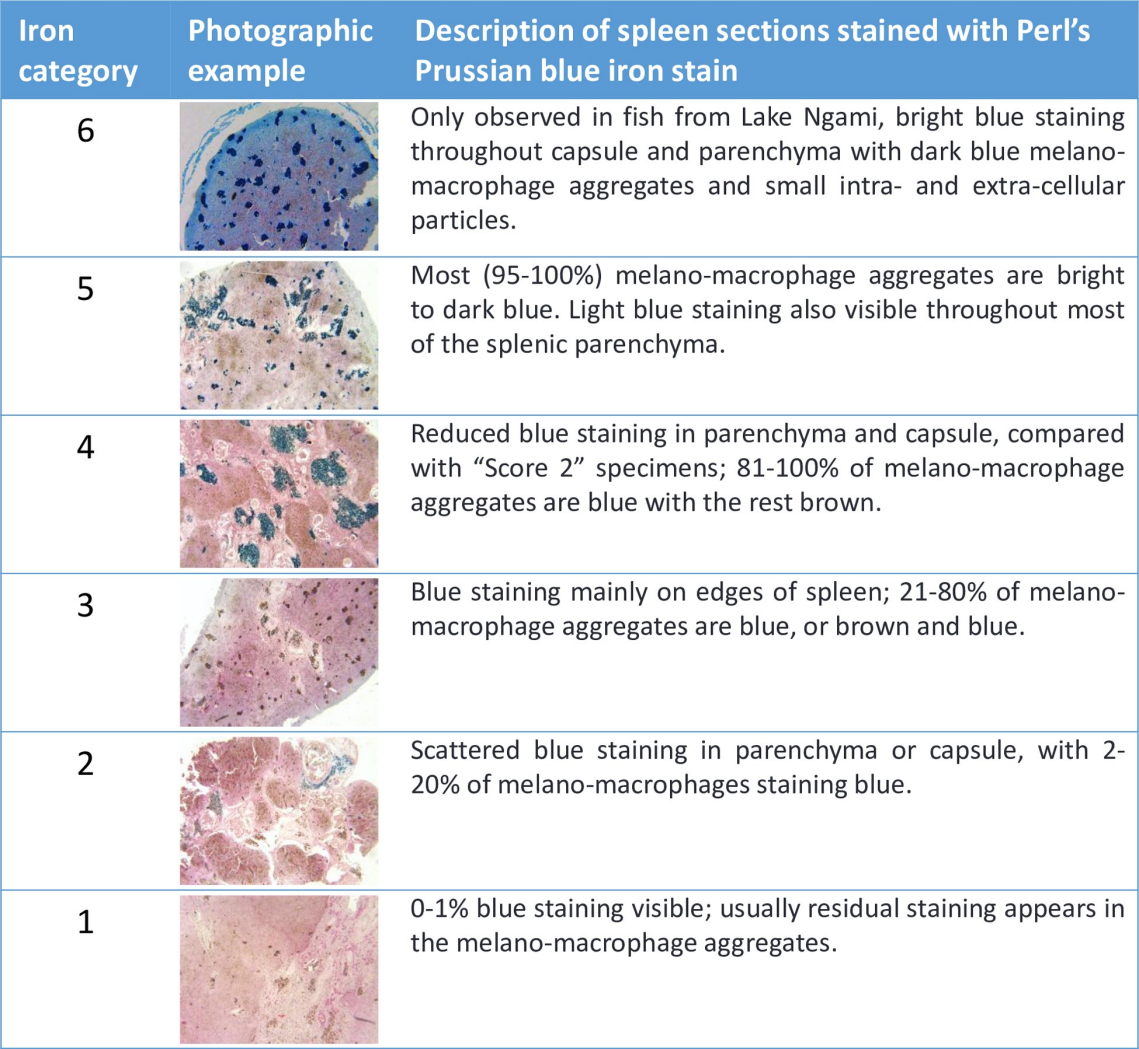
Iron category definitions for spleen sections stained with Perl’s Prussian blue iron stain. Categories 1–6 reflect the degree of iron staining in spleen sections, with 6 representing the most iron. Category 6 was only observed in Lake Ngami fish. Higher resolution images are available in S2 Fig.

**Fig 4:**
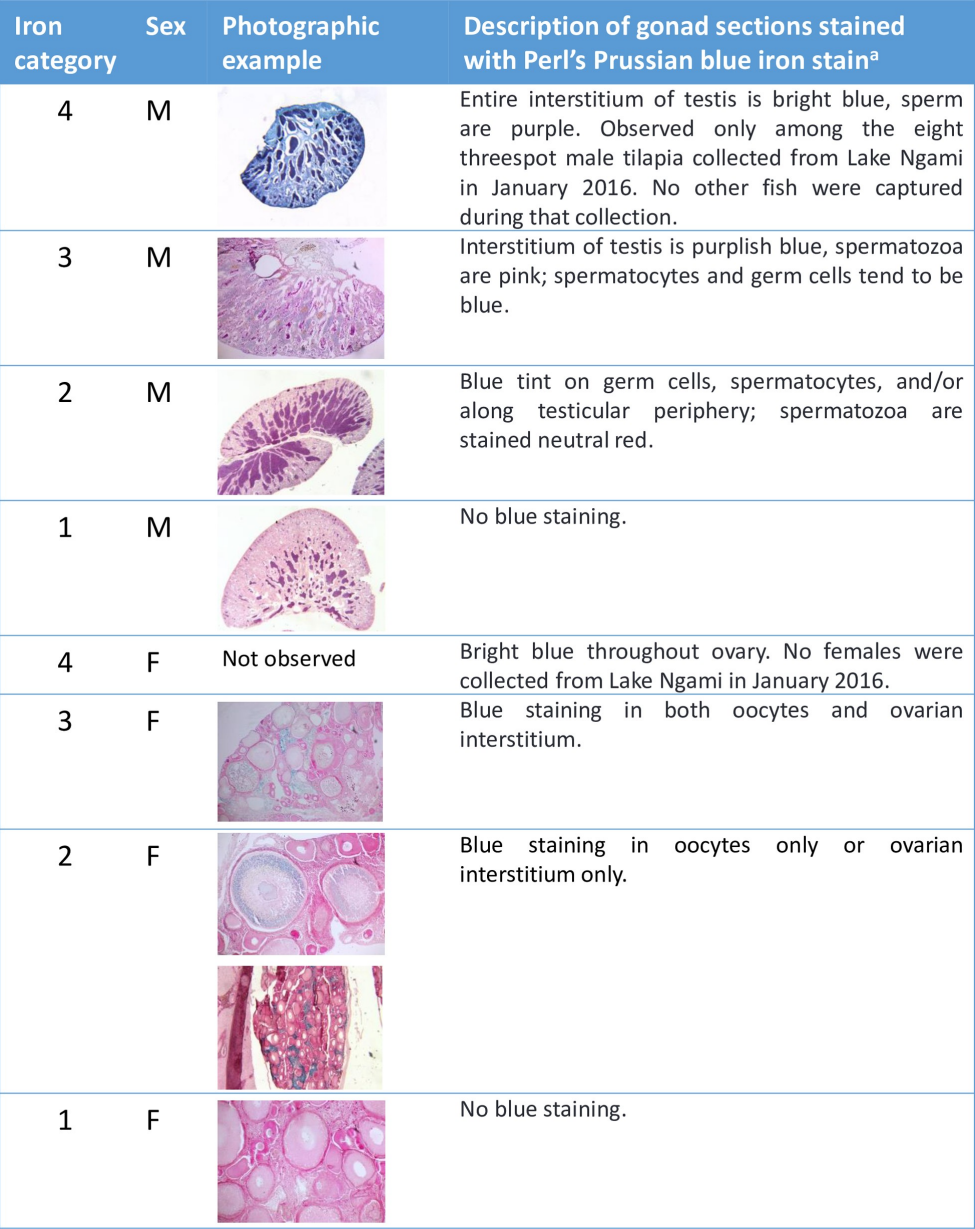
Iron category definitions for gonad sections stained with Perl’s Prussian blue iron stain. Categories 1–4 reflect the degree of iron staining in testis and ovary sections, with 4 representing the most iron. Category 4 was only observed in Lake Ngami fish, which were all male. Unlike liver and spleen, gonadal iron staining was infrequently observed and less abundant, with little evidence of concurrent inflammation. Additionally, among females, gonadal iron, if present at all, appeared to depend on oocyte developmental stage, with iron moving from the interstitium into oocytes as the oocytes developed. For these reasons, we did not include gonadal iron in the more extended pathology analysis. Higher resolution images are available in S3 Fig.

Fixed samples were transported back to the United States in 10% formalin, transferred to 70% ethanol, dehydrated through graded ethanol solutions, infiltrated with paraffin wax for four hours in a vacuum oven at 56°C, embedded, sectioned at 7 μm, and stained with hematoxylin and eosin (H&E) [14]. Toluidine blue staining distinguished mast cells from other granulocytes [15–17]. To quantify iron accumulation in livers, spleens, and gonads, we stained additional sections with Perl’s Prussian blue [18] and developed a scoring index, defined in Figs 2–4. Geographic and seasonal variation in hepatic and splenic iron content was visualized in a mosaic plot and assessed statistically using Pearson’s chi square.

Similarly, inflammation and cellular damage in liver, spleen, and gonad tissues was scored and categorized as described in S2 Table. Geographic and seasonal variation in hepatic and splenic inflammation was also visualized in a mosaic plot and assessed statistically using Pearson’s chi square. For all liver and spleen sections, we noted the color of the granulocytes and other pathological features that occurred in the samples, including vacuoles, hyaline droplets, white spaces, parasites, fat cells, lymphocytes, fluid-filled cysts, congested blood vessels, occluded splenic ellipsoids, and hepatocytic glycogen depletion. Finally, histological gonad stage was determined for males by noting the presence/absence of spermatogonia, spermatocytes, spermatids, and spermatozoa, and for females by measuring the diameter of the largest oocyte in the section under the 10x objective of the microscope.

### Aquaculture comparison samples

To obtain reference tissues with normal histology, we collected samples from Nile-blue tilapia hybrids (*Oreochromis niloticus/aureus*) (n = 5) raised in aquaculture facilities at the University of Georgia (UGA), Athens, Georgia. UGA fish were fed a diet containing supplementary iron and they exhibited elevated splenic iron in some individuals, suggesting that tilapia sequester excess iron in their spleens as shown for many fish species [19]. This feature is relevant to the interpretation of the present study. To compare the effects of salt-drying and oven-drying on heavy metal content in muscle and liver tissues, duplicate muscle and liver samples were taken from each UGA fish and processed as described above. Additionally, we obtained samples from three-spot tilapia (*Oreochromis andersonii)* (n = 3 for liver, 2 for spleen) from a small aquaculture facility (Hatty Farm) in Pandamatenga, Botswana. Samples were processed similarly to those from the Okavango fishes.

### X-ray fluorescence (XRF) spectroscopy

Oven- and salt-dried liver and muscle samples from all Okavango tilapia, along with salt- and oven-dried samples from UGA (n = 5), were milled to a fine powder at 50 Hz for 2 minutes; 0.5 g of each sample was pressed into a 1 cm diameter mold under consistent pressure to produce 0.5 mm thick pellets. Using a Bruker Tracer III-SD, we evaluated heavy metal burdens in pellets from all sampled fish. Measurements were collected at 40 keV and 10 mA for 360 seconds, without an additional filter allowing measurement of all elements ranging from Mg to U.

### Data analysis

Data were managed in Microsoft Excel. Water quality data from loggers and handheld devices were plotted using Excel. All statistical analyses were completed using JMP 14 (SAS Institute, 2018, Cary, North Carolina, United States). Frequencies of inflammation and iron accumulation among fish were compared across collections using Pearson’s chi square analyses and visualized in mosaic plots. Mean spleen-somatic index (SSI) of fishes from each collection were compared using one-way Welch’s analysis of variance (ANOVA) for samples with unequal variances, followed by Games-Howell multiple comparisons. Differences among groups were considered significant when p ≤ 0.05.

## Results

### Water quality

**Fig 5:**
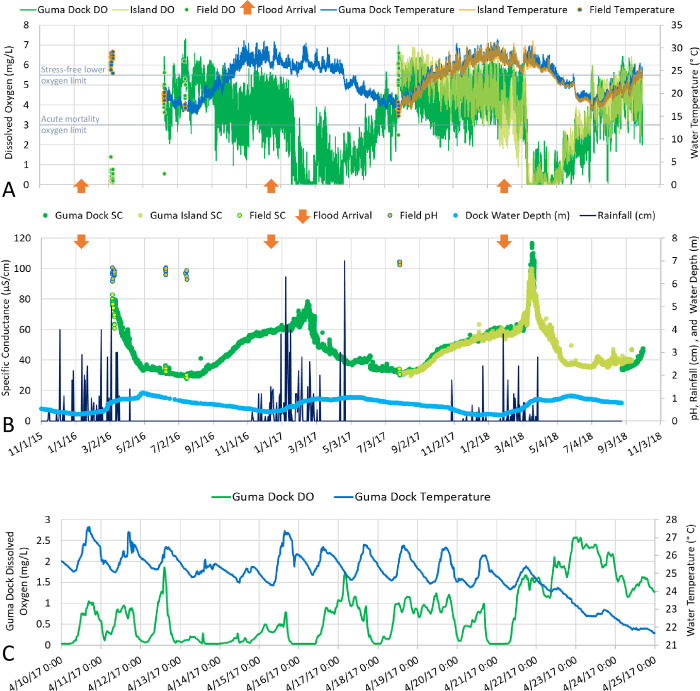
Abiotic conditions in Guma Lagoon. Dissolved oxygen (DO) and temperature collected with data loggers and by hand (“field”) at Guma Lagoon Camp dock and on a tree island approximately 3 km downstream on the opposite side of Guma lagoon (A). Similarly collected specific conductance (SC) and field pH are shown in (B). Orange arrows indicate flood arrival, based on first observed rise in water level. Water depth at Guma Lagoon Camp dock and rainfall (B) are monitored daily at Guma Lagoon Camp. Example daily resolution data for water temperature and DO during post-flood hypoxia (C) show that temperature followed an expected diurnal pattern with lows occurring just before sunrise and highs in the mid to late afternoon. The daily DO pattern was less predictable, sometimes tracking with temperature, but not always. At times, both peaks and troughs in DO levels were observed during the night and from midday to late afternoon. This variation suggests that photosynthesis was not the main determinant of DO. Ecosystem respiration likely outpaces photosynthesis in terms of oxygen levels during the hypoxic periods [2].

Okavango water quality was ultra-fresh with conductivity values as low as 23 μS/cm in the Panhandle, and rising as water moves downstream, to Nxaraga (82–142 μS/cm), Xakanaxa (76–88 μS/cm), Chanoga (142–300 μS/cm), and Lake Ngami (400–538 μS/cm), with significant variation depending on season and flood water volume (S1 Table and Fig 5). At Guma, the flood brought elevated tannins and solute loads that caused a 2.7 to 3.7-fold increase in aquatic conductivity (Figs 1E and 5). This rise in conductivity then declined as water levels rose and salts were diluted (Fig 5). Water temperature varied seasonally from 16 to 32°C and was consistent (generally within 1°C) throughout the water column in the channel and lagoons, due to the shallowness of the Delta (rarely deeper than 4 meters) (S1 Table and Fig 5). Like conductivity, pH increased slightly from north to south, ranging from 6.1 to 6.7 at Guma, 6.6 to 7.8 at Xakanaxa and Nxaraga, and 8.7 to 9.4 at Chanoga (S1 Table and Fig 5). Arrival of tannic flood waters was usually accompanied by a decrease in pH, particularly in the downstream areas. Nitrate and nitrite concentrations were usually zero, with occasional measurements of 1 to 2 mg/L NO_3_-N and up to 0.03 mg/L NO_2_-N (S1 Table).

As expected, dissolved oxygen levels in the Delta followed a seasonal pattern defined by flood dynamics. Based on Guma logger data from 2016–2018, hypoxia initiated in the lagoon approximately 45 days after flood-water levels began to rise and persisted for 3.5 to 5 months (Fig 5). During the hypoxic period, DO at Guma rarely exceeded 3 mg/L and periodically persisted below 0.5 mg/L for up to two weeks (Fig 5). Therefore, tilapia sampled during the hypoxia were surviving at DO levels that were well below those expected to support life [13]. In addition to being long-lasting, the band of hypoxia moved slowly through the Delta. For example, in March 2016, the band, characterized by uniform hypoxia and tannins, was detectable at Guma and the Duba Islands, but would not reach Nxaraga until May (S1 Table).

In addition to seasonal variation in dissolved oxygen concentrations, there was diurnal variation, with DO changing by up to 2 mg/L within the day. Interestingly, daily DO patterns tracked with temperature and sunrise/sunset sometimes, but not always. At times, both peaks and troughs in DO levels were observed during the night and from midday to late afternoon (Fig 5). This variation suggests that photosynthesis was not the main determinant of DO. Instead, ecosystem respiration likely outpaces photosynthesis in terms of DO levels during the hypoxic periods [2].

### Fish demographics

**Fig 6:**
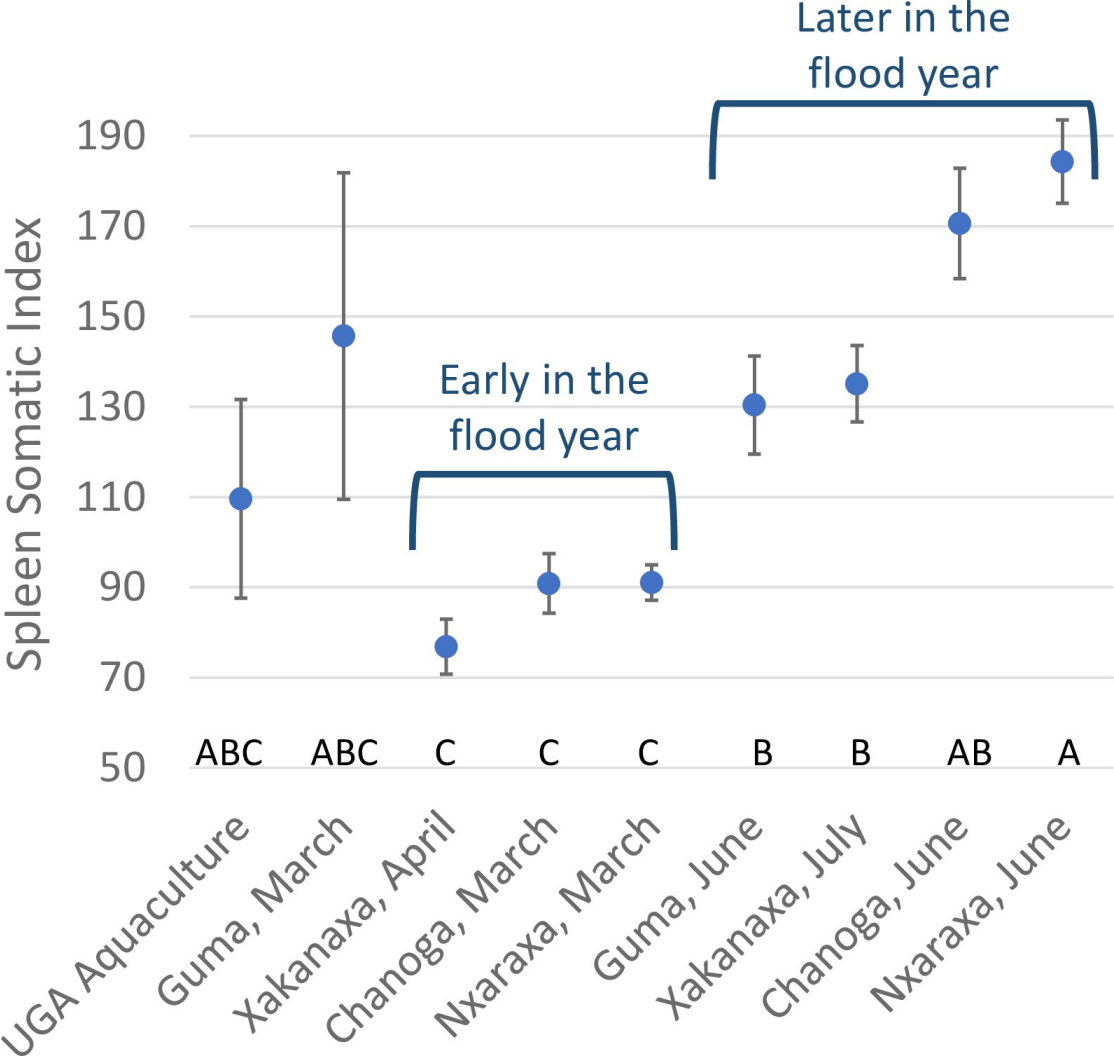
Mean (+/- 1 SE) spleen-somatic index (SSI) was elevated in fish collected later in the flood season, compared with fish collected earlier in the year, before hypoxic conditions began. Data suggest that hypoxic exposure is associated with splenomegaly, as is expected during hypoxia-induced polycythemia. SSI = ((spleen weight (mg)/fish weight (g))*100). Collections not connected by the same letter at the bottom of the graph are significantly different, based on one-way Welch’s analysis of variance (p < 0.0001) for samples with unequal variance, followed by Games-Howell post-hoc comparisons.

Catch rates for each of the three species of fish varied by site and time of year. Adult *Oreochromis andersonii* females and males were captured at all sites and times, except Lake Ngami, which was sampled just once and yielded only males (S1 Table). We made several attempts to fish Lake Ngami on additional occasions, but low water associated with drought conditions hindered boat access. Likewise, adult *Coptodon rendalli* and *O*. *macrochir* males and females were captured at all sites except Lake Ngami during at least one sampling visit, but with smaller sample sizes than *O*. *andersonii* (S1 Table). Size ranges and other morphometric data are provided in S1 Table. Although fish exhibited hepatic and splenic pathologies, they appeared to be reproducing normally, with all stages of seasonally appropriate gamete production and gonad development represented. Signs of gonadal inflammation were generally minor or non-existent, so we focused our analytical efforts on the liver and spleen. Of the three organ-somatic indexes measured, only spleen-somatic index (SSI) differed significantly among collections, with SSI increasing at Nxaraga, Xakanaxa, and Chanoga as the flood season progressed (Fig 6).

### Inflammation and cellular damage

#### Liver

**Fig 7:**
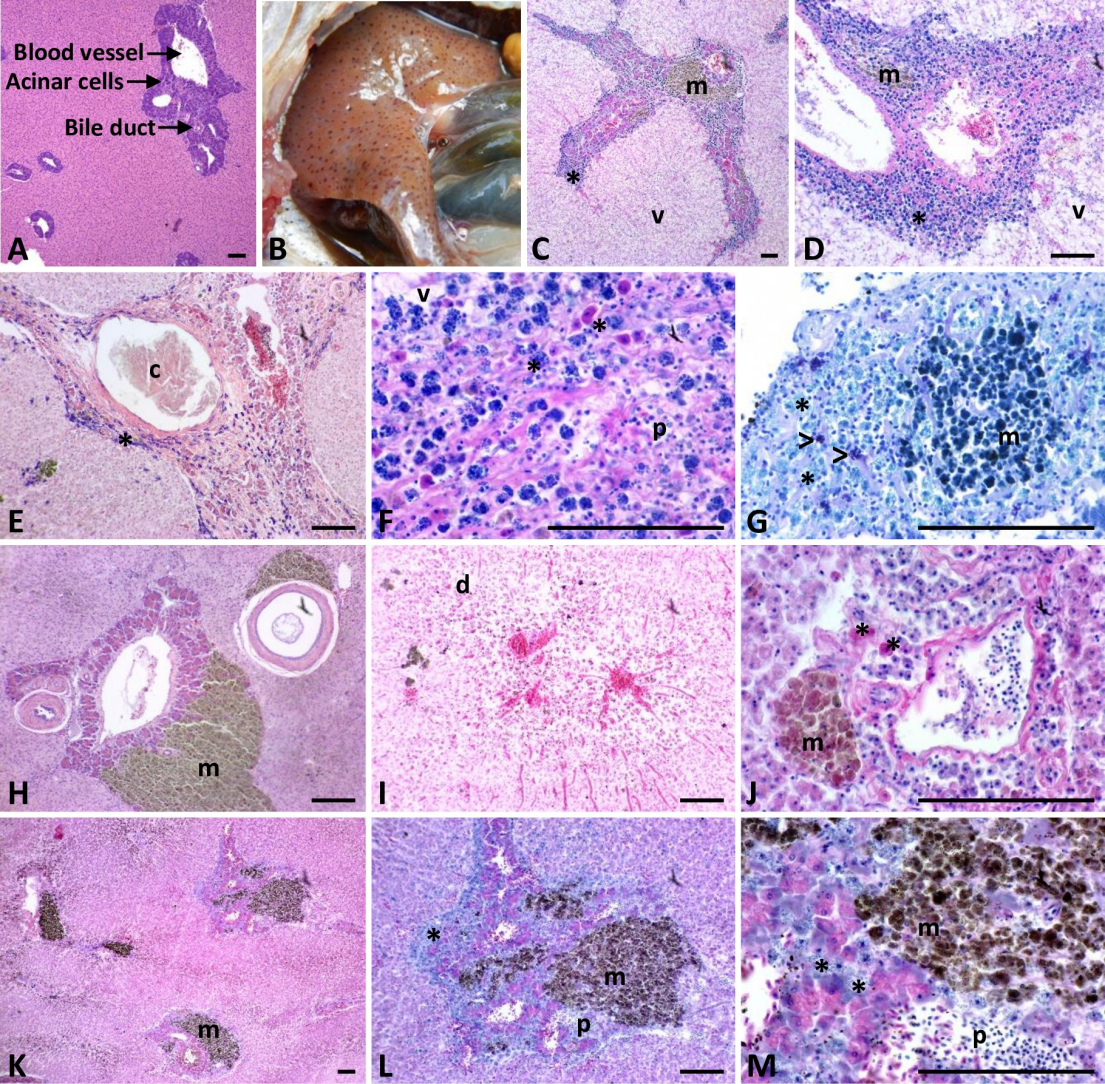
Normal and pathologic hepatic histology. Normal liver histology for *Oreochromis mossambicus* collected in South Africa (A) followed by representative hepatic inflammatory pathologies observed in three species of Okavango tilapia: *Oreochromis andersonii* (B-G), *Coptodon rendalli* (H-J), and *Oreochromis macrochir* (K-M). Sequentially magnified images of the same histological section are shown in K-M. Macroscopically, melanomacrophage aggregates appear as pigmented dots on the liver (B). Inflammation was characterized by species-specific aggregations of basophilic (C-G, K-M) or eosinophilic (F, J) granulocytes (*) that surrounded acinar cell clusters. Some individuals exhibited both basophilic and eosinophilic granulocytes, such as the threespot tilapia shown in (F). Purple mast cells (>) were distinguished from other granulocytes (*) by staining with toluidine blue (G). Other common pathological features, observed in all three species, included thickened blood vessel and bile duct walls (D, E, H), fibrosis (D, E, F, J), dilated or congested blood vessels and sinusoids (I), large melanomacrophage aggregates (m) (C, D, G, H, J-M), and varying degrees of brown, intracellular ceroid pigment accumulation within hepatocytes (E, H, I, K, L). In more advanced cases, fibrosis worsened and was associated with fluid filled hepatic cysts (c) (E), lymphocytic infiltration (p) (F, L, M), vacuolar degeneration (v) (C, D, F), glycogen depletion (D), and cellular destruction (d) (I) that originated within acinar bundles and then moved out to surrounding hepatocytes, resulting in total loss of acinar clusters (D, E, F, I, J). All scale bars are 100 μm. Higher resolution images are available in S4 Fig.

**Fig 8:**
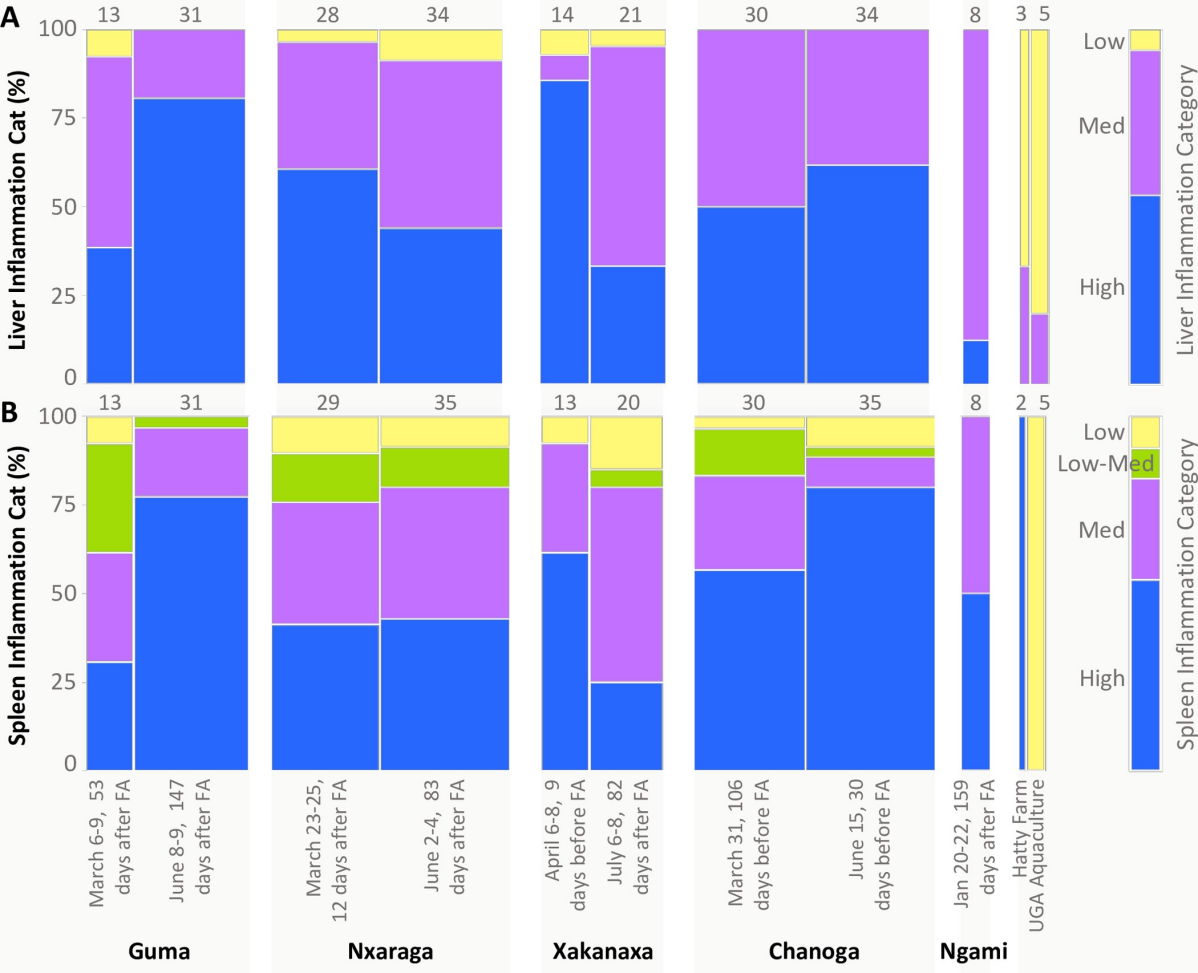
Mosaic plot comparing the percentages of fish from each collection that exhibited varying degrees of inflammation and cellular damage in liver (A) and spleen (B). Collections are presented chronologically for each site in relation to the timing of flood arrival (FA) and predicted decline in dissolved oxygen. The ranges of dissolved oxygen concentrations measured during the collection are shown in blue font on the x-axis, although it is important to recognize that these measurements represent a point in time during the highly dynamic flood season (DO means and sample sizes provided in S1 Table). Inflammation categories are defined in S2 Table. The width of each column within the plot is proportional to sample size; total sample sizes for each collection are also provided above each column. Data from comparative farmed fish, from “Hatty Farm” and “UGA Aquaculture,” are also shown.

Although fish appeared healthy at capture, histological liver analysis revealed medium or high levels of inflammation, cellular damage, and fibrosis in 94% of sampled fish (n = 221) (Figs 7 and 8A and S2 Table). Normal tilapia liver (functionally a hepatopancreas) consists of bundles of exocrine pancreatic acinar cells, clustered around blood vessels or bile ducts, and embedded within the hepatocytic parenchyma (Fig 7A). Additionally, in healthy fish, granulocytes occur sparsely in many tissues and play a monitoring and phagocytic role as first-line, immunological defenders [20]. We confirmed sparse hepatic granulocytes in four of the five control samples from UGA Aquaculture-raised *Oreochromis niloticus*-*aureus* hybrids. But, granulocyte numbers escalate, along with lymphocytes and macrophages, when fish are challenged by pathogens, parasites, contaminants, or hypoxia [21–23]. Hence, in the Okavango fish, we observed profuse aggregations of mast cells and granulocytes, both intact and degranulating, surrounding most or all acinar cell clusters (Figs 7 and 8A).

Interestingly, the hematoxylin and eosin staining characteristics of granulocytes varied by genus in this study (Fig 7). In threespot and greenhead tilapia, most granulocytes were basophilic and abundant, although small numbers of eosinophilic granulocytes with blue or pink granules and double lobed nuclei (neutrophils) were also sometimes observed. In redbreast tilapia, granulocytes were usually eosinophilic and more moderate in number, although 10% of sampled redbreast tilapia exhibited basophilic granulocytes with occasional eosinophilic nuclei. In fishes, leukocyte staining characteristics have been established to vary by genus and their influx is frequently associated with chronic inflammation [15, 16].

In addition to granulocytic infiltration in the livers of Okavango tilapia, the walls of hepatic blood vessels and bile ducts were frequently thickened or replaced by fibrosis (Figs 7 and 8A and S2 Table). In more advanced cases, fibrosis worsened and was associated with lymphocytic infiltration, pyknotic nuclei, glycogen depletion, vacuolar degeneration of hepatocytes, and necrosis. In a manner similar to that reported for autoimmune pancreatitis [24], cellular disruption and destruction originated within acinar bundles but moved out to surrounding hepatocytes after acinar bundles became necrotic, apoptotic, or replaced with fibrosis (Fig 7). Hyaline droplets and vacuolation were sometimes observed and fluid-filled hepatic cysts were common (Fig 7). Finally, acinar bundle inflammation was often associated with large melanomacrophage aggregates (MMAs) with varying degrees of hemosiderin, ceroid, or lipofuscin accumulation within the cytoplasm of hepatocytes (Fig 7).

#### Spleen

**Fig 9:**
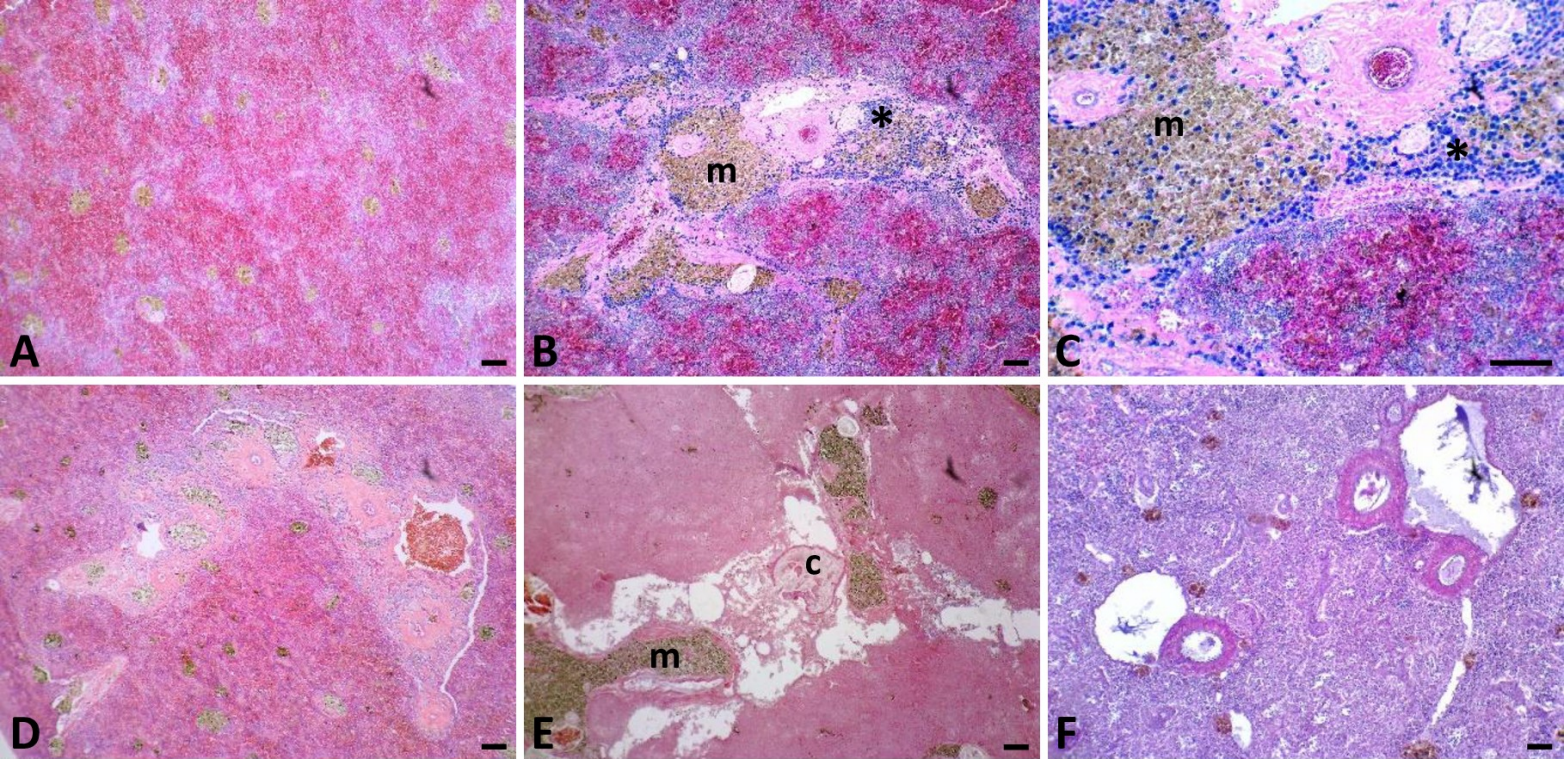
Normal and pathologic splenic histology. Normal splenic histology for *Oreochromis andersonii* (A) followed by representative splenic inflammatory pathologies observed in three species of Okavango cichlids. Sections shown are from *Oreochromis andersonii* (B-E) and *Coptodon rendalli* (F), including sequentially magnified images of the same histological section (B, C). Inflammation was characterized by varying degrees of granulocytic infiltration (*), thickened blood vessel walls (B-D, F), congested blood vessels (D), fibrosis (B-E), cellular disruption and destruction, vacuolation (E, F), fluid-filled cysts (c) (E), and increased number and size of melanomacrophages (m) (B-F). All scale bars are 100 μm. Higher resolution images are available in S5 Fig.

Concomitant with hepatopancreatic inflammation and cellular damage, splenic inflammation was also widespread, affecting 91% of all sampled fish (Figs 8B and 9 and S2 Table). Additionally, the degree of hepatic and splenic inflammation among individual fish was significantly correlated (p < 0.0001). Inflamed spleens showed varying degrees of granulocyte infiltration, thickened blood vessel walls, thickened and occluded ellipsoids, fibrosis, hyaline droplet accumulation, cellular disruption and destruction, vacuolation, and the presence of fluid-filled cysts (Figs 8B and 9). Although MMAs are common findings in normal fish spleens (Fig 9A), their number and size were increased in most Okavango fishes, along with often substantial intracellular ceroid and/or lipofuscin accumulation (Figs 8B and 9 and S2 Table).

#### Relationship between inflammation/cell damage and timing of the flood and associated hypoxia

The mosaic plot shown in Fig 8 and associated Pearson’s chi-square indicate that the distribution of inflammation categories differed significantly across collections (p <0.0001 for both liver and spleen). At Guma, the only site where we collected continuous oxygen data, inflammation severity in livers and spleens increased as the hypoxic season progressed between March and June. An opposite trend occurred at Xakanaxa, in association with more rapid oxygen recovery after flood arrival (Fig 8). Although hypoxic conditions were detected at Nxaraga during both collections, we observed some reduction in liver inflammation severity between March and June, while spleen inflammation frequency remained unchanged. At Chanoga, fish exhibited marked inflammation and cellular damage during both collections, which were both taken well before flood arrival, during times of normoxia. We were not able to stay in Botswana long enough to sample fish after the flood reached Chanoga. With the exception of one fish, the control fish from UGA exhibited no or low levels of inflammation or cellular damage in livers and spleens. Oxygen levels in the UGA tanks averaged 6.3 mg/L during our visit. The fish from Hatty Farm in Botswana did exhibit elevated splenic pathology and one of three fish had medium hepatic inflammation. Oxygen levels in the Hatty Farm tanks were around 4 mg/L at mid-day during our visit. These values are low but not hypoxic.

### Iron accumulation

**Fig 10:**
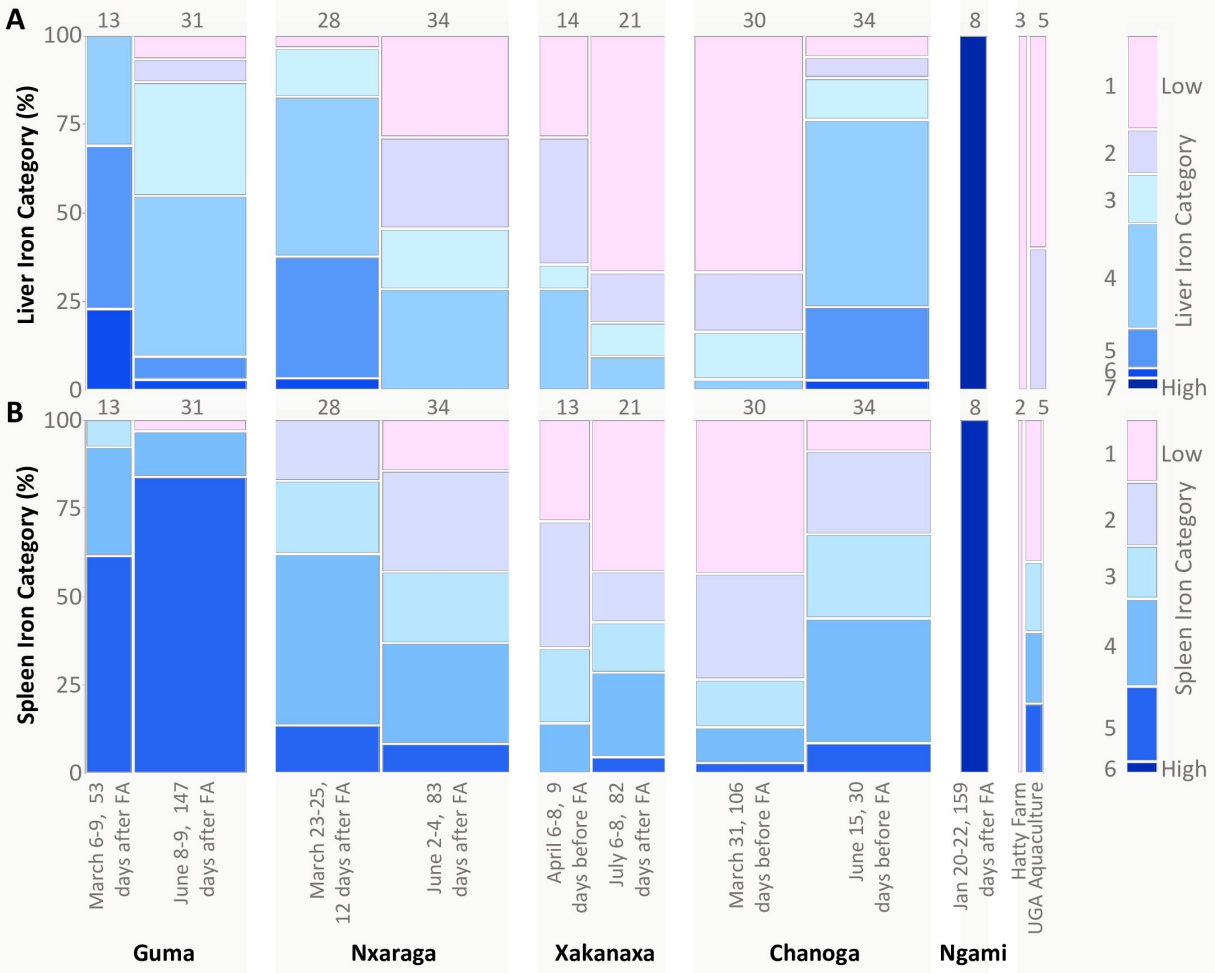
Mosaic plot comparing the percentages of fish from each collection that exhibited iron staining category 1–7 for liver (A) and category 1–6 for spleen (B). Collections are presented chronologically for each site in relation to the timing of flood arrival (FA) and predicted decline in dissolved oxygen. The ranges of dissolved oxygen concentrations measured during the collection are shown in blue font on the x-axis, although it is important to recognize that these measurements represent a point in time during the highly dynamic flood season (DO means and sample sizes provided in S1 Table). Iron categories are defined in Figs 2 and 3, with “1” representing the lowest level of iron deposition. The width of each column within the plot is proportional to sample size; total sample sizes for each collection are also provided above each column. Data from comparative farmed fish, from “Hatty Farm” and “UGA Aquaculture,” are also shown.

Perl’s Prussian blue staining of liver and spleen samples indicated that melanomacrophage aggregates and associated granules of brown pigment were often iron-rich (Figs 2–4 and 10) and the presence of elevated iron concentrations was confirmed for Lake Ngami fish using x-ray fluoroscopy (see details below). In comparison with UGA and Hatty farmed tilapia, fishes with elevated hepatic iron were observed at all Okavango sites, with significant variation among sites and collection dates (p < 0.0001), suggesting a seasonal factor in iron accumulation (Fig 10A). Additionally, elevated iron was significantly correlated with increased inflammation in liver tissues (p = 0.048); a similar trend was observed for spleen samples, but the correlation did not reach significance (p = 0.069).

#### Liver

The highest levels of hepatic iron occurred in fishes from Lake Ngami in January, Guma in March and June, Nxaraga in March, and Chanoga in June (Fig 10A). At Guma, more than the other sites, sampling coincided most precisely with flood-induced hypoxia, which established at the end of February, an estimated 45 days after flood arrival (Fig 5A). The Guma data suggest that hypoxia was a driver of iron accumulation, which then declined in the liver, as DO recovered post-flood. This pattern was repeated at Nxaraga and Xakanaxa. But, at Chanoga, which is far downstream from the other sites, we observed greatly elevated hepatic iron during June, compared with March, even though the flood and predicted hypoxia would not arrive for another month at least.

#### Spleen

Fish from UGA Aquaculture (n = 5) exhibited the full range of spleen iron categories but without associated inflammation or iron accumulation in the liver, suggesting that the spleen is preferred over the liver as an iron storage site and that excess iron storage is normal for the spleen, as has been shown for many fish [19]. UGA fish were also fed an iron-enriched diet, which could contribute to splenic iron storage. Hatty Farm fish (n = 2) did not exhibit any accumulated iron in spleen tissues. We note that sample sizes for UGA and Hatty Farm were low. Compared with UGA and Hatty Farm fish, elevated splenic iron was observed in fish from Lake Ngami and Guma, and to a lesser extent at Nxaraga for both collections and Chanoga in June. At Guma, the marked elevation in splenic iron observed in March was even more prevalent in June, despite increasing oxygen, flood recovery, and declining hepatic iron (Fig 10B).

### X-ray fluorescence to evaluate heavy metals

During fieldwork, liver and muscle samples were dried in salt to preserve them for later heavy metal analysis. However, later testing revealed that salting removed 80–100% of the metals, which were presumably and unintentionally discarded with the brine, and rendered all samples un-usable for heavy metals analysis, except those from Lake Ngami, which were oven-dried. In the Lake Ngami samples, iron concentrations were significantly higher (100-fold) in liver compared to muscle (p < 0.0001). Whereas, in oven-dried samples from the UGA fish, the difference between liver and muscle iron was just 6-fold higher in liver (p < 0.0001). Moreover, compared with UGA fish, Lake Ngami fish livers contained significantly more iron (24-fold higher), but muscle iron content was similar. Other elements qualitatively identified in Okavango fish from all sampled sites included nickel, copper, arsenic, selenium, and zinc, but none of these were elevated in Lake Ngami fish compared with UGA fish.

## Discussion

In this study, we report dramatic levels of inflammation and iron accumulation in livers and spleens of three fish species sampled throughout the Okavango Delta during the annual flood season. This is a new, and quite unexpected discovery in the Okavango, and as such, it is important to determine the cause. Our data and the work of others indicate that the flood brings marked changes in water conductivity, pH, and dissolved oxygen [25]. Most notably, as the leading edge of the flood traverses the Delta, it brings with it a deep band of hypoxia that lingers for months in the Panhandle. In the more southern reaches of the Delta, the hypoxic band rolls through, but possibly with more variability [25].

At Guma, where March sampling coincided most closely with arrival and progression of hypoxia, we observed elevated iron accumulation that seems to precede increased inflammation in the liver and spleen of resident cichlids. Moreover, fish collected at Guma in March exhibited much greater variance around the mean SSI, suggesting the population was in a transitional state, as the hypoxia and flood had arrived at Guma just prior to the fish collection. As we moved downstream, to Nxaraga and Xakanaxa, the pattern of iron accumulation repeated, although the over-all degree was less than that observed at Guma. It is possible that we missed peak iron accumulation at Nxaraga and Xakanaxa, or the duration of hypoxia was less extreme than at Guma. This consideration is supported at Nxaraga, where Cawley et al (2012) reported that channel water mixes dynamically with water in adjacent flood plains and wetlands, and the mixing may hasten DO (and iron) recovery [2]. In comparison, the river at Guma is more channelized and a lack of water mixing may delay DO recovery. Further downstream, at Chanoga in June, we observed elevated tissue iron concentrations in fish even before flood arrival. This could occur if hypoxia-exposed fish with high iron loads escaped downstream and joined the sampled population at Chanoga. We did not sample Chanoga after flood arrival, so direct effects of the flood and any associated hypoxia on resident fish at Chanoga are currently unknown.

The observations at Chanoga highlight several additional factors that could affect the etiology and timeline of the observed pathology but have not yet been sufficiently characterized: site differences in mixing, water flow rate, interactions with adjacent wetlands, fish movement, and fish age. Considered together, these variable factors suggest that hypoxia exposure is a dynamic and moving target in the Delta and, therefore, variation of these factors among sites should result in variable fish responses. Additionally, the progression, duration, and regression of the observed pathology is completely unknown. So, after the hypoxia passes, we do not know how long it takes for fish to recover or even if they do. From our limited observations, accumulated iron does appear to lessen in tissues over time, with spleen recovery lagging behind liver. However, the reduction may not be absolute. Additionally, a spleen recovery lag may be related to the spleen’s normal role as an iron storage depot [19, 26]. Moreover, the cycle of iron accumulation and dissipation from the liver and spleen likely repeats with each flood season, potentially creating cumulative stress pathologies associated with iron excess that would be most evident in older fish. The presence of non-reversible fibrosis in many fish in this study suggests that fish accumulate permanent damage over time.

As shown in Figs 2–4 and 10, the highest iron loads were detected in Lake Ngami fish collected in January 2016, following the 2015 flood season. How fish from Lake Ngami fit broader patterns for the Okavango is difficult to assess because water quality in the lake differs substantially from the rest of the Delta. In 2015–2016, the flood did not reach Lake Ngami and the lake became isolated and subject to considerable drying, concomitant with elevated conductivity (~400–538 μS/cm) and pH (8.7–9.4). We lack oxygen data collected in 2015 or early 2016 for the lake. However, daytime field measurements obtained from monitoring records in 2008–2010 and our measurements taken in mid-2016 and 2017 indicate DO concentrations between 1.76–13.7 mg/L. This wide variation does not occur in the rest of the Delta and is likely due to high phytoplankton loads supported by nutrient inputs from livestock, which use the lake in higher numbers than other areas of the Delta that are mainly protected as national parks. Additionally, an unpublished 2008–2010 field survey of dissolved iron, performed along a transect in the Delta by staff at the Okavango Research Institute, indicated that iron in Lake Ngami varied from <0.1 to 1.86 mg/L, while the maximum dissolved iron upstream in Maun was 0.25 mg/L. These data suggest there is significant iron enrichment in Lake Ngami that likely influences iron metabolism in the resident fish. For these reasons, the fish in Lake Ngami could be considered separately from fish in the main Okavango Delta.

The hypothesis that hypoxia caused the observed pathologies is supported by several lines of evidence. Among vertebrates, fishes exhibit especially diverse responses to hypoxia because aquatic habitats are vulnerable to substantial variation in oxygen availability. These include mechanisms that increase oxygen uptake (increased gill surface area and surface breathing), promote oxygen delivery (increased red blood cell production and blood vessel proliferation), decrease metabolic demands, and favor anaerobic metabolism [27]. For example, in East Africa’s Lake Victoria, cichlids exposed to hypoxia improve oxygen transport by increasing hemoglobin-oxygen affinity [28]. These physiological responses are regulated by hypoxia signaling pathways that are strongly conserved across animal taxa [29]. In addition to increased red blood cell production, mammals and fishes also elevate circulating leukocyte numbers (neutrophils and other granulocytes) in response to hypoxia [28, 30–32]. These hematological changes are common in fish exposed to hypoxia as well as in tobacco smokers and people or livestock living at high altitude [33–35]. Both smoking and high altitude living recapitulate aspects of chronic hypoxia in terrestrial environments.

Erythropoietin (Epo) is one key hypoxia-induced gene that promotes hemoglobin, red blood cell production, and associated elevations in hematocrit. Epo has been studied in several fish species, including pufferfish (*Takifugu rubripes*), rainbow trout (*Oncorhynchus mykiss*), carp (*Ctenopharyngodon idellus*), and eel (*Anguilla anguilla*) (reviewed by [29]). Other important master genes in the hypoxic responses of fishes (and other vertebrates) include hypoxia-inducible factors (at least three isoforms of Hif-α and Hif-1β), insulin-like growth factor binding protein (igfbp), neuronal nitric oxide synthase (nNOS), and protein carbonyl (PC) [36, 37]. In both adults and larvae of multiple species, including Atlantic croakers (*Micropogonias undulates*), zebrafish (*Danio rerio*), and Nile tilapia (*Oreochromis niloticus*), low oxygen induces elements of hypoxia signaling pathways within a few hours of exposure across diverse tissues, including liver, brain, eye, heart, spleen, gill, testis, kidney, swim bladder, and red blood cells [36–38].

These findings illustrate that elevated red blood cell production (polycythemia) and associated iron recycling are common responses to hypoxia in fishes, as they are in mammals. In both groups of vertebrates, long-lasting hypoxia-induced polycythemia can lead to splenomegaly, arterial congestion, blood vessel wall thickening, increased hemolysis, and/or hemochromatosis [34, 39–42]. With increased red blood cell recycling, the resulting iron excess can accumulate as hemosiderin in the liver, pancreas, and spleen where it can induce nonspecific inflammation and fibrosis [41, 43–45]. As part of the inflammatory response, the accumulated iron attracts macrophages that remove excess red blood cells, promote iron recycling, and sequester iron, all in an effort to defend tissues against iron overload and related cellular toxicity [21, 46, 47].

In fishes, iron rich macrophages bundle into easily viewed melanomacrophage aggregates (MMAs) in the liver, head kidney, and spleen, and these were frequently macroscopically visible in the livers of the Okavango fishes (Fig 7B). Together with hemosiderin, MMAs typically contain ceroid and lipofuscin (lipoproteins that accumulate in the wake of cellular destruction and necrosis), and melanin, which aids in the removal of cellular debris [21, 41, 48]. To enhance their management of cellular damage, MMAs tend to attract granulocytes and lymphocytes and are frequent in fishes affected by chronic inflammation [21, 48]. Although MMAs naturally increase with age, MMA abundance in fish organs is considered a reliable biomarker of fish stress caused by aquatic hypoxia and/or chemical pollution [21, 41, 48].

Many of the outcomes associated with chronic hypoxic exposure, including inflammation, granulocyte infiltration, cellular damage, arterial congestion, blood vessel wall thickening, formation of abundant MMAs, iron accumulation, and splenomegaly, were observed in the Okavango cichlids in this study. These features suggest that the Okavango fish survive extended hypoxia through a polycythemic mechanism; the increased red blood cell production leads to multi-organ hemochromatosis, which in turn, stimulates inflammation, tissue destruction, and fibrosis. The observed inflammation may also be a direct response to hypoxia itself [49]. Hypoxia is a notable cellular feature of many pathological processes that attract granulocyte and macrophage infiltration, including cancer, wound healing, and atherosclerosis [50]. Thus, these findings in Okavango fishes are aligned with more universal vertebrate responses to tissue hypoxia and iron toxicity. In future studies, data on hematocrit and blood hemoglobin concentrations would be essential for testing this hypothesis.

Although hypoxia clearly impacts Okavango fish health, the presence of several genus-specific granulocyte cell types showing variable staining characteristics suggests more than one underlying cause of the observed inflammation. Elevated granulocyte numbers were previously reported for Okavango threespot tilapia, collected in June under normoxic conditions in the Panhandle [22]. In that study, 60% of fish (n = 15) exhibited acinar bundle granulocyte invasion and hepatopancreatic steatosis in association with elevated parasite loads. In our collections, parasites were present in some fish, but were not required for the observed pathologies. None of our fish exhibited hepatopancreatic steatosis. Moreover, the granulocytes observed in the previous Panhandle study were distinctly eosinophilic [22], whereas our threespot tilapia samples presented primarily basophilic granulocytes. These differences suggest that the cause of inflammation in our study was distinct from the scenario previously reported in the Panhandle [22].

Although hypoxia is clearly a significant factor in Okavango ecology, it is not the only water quality factor that could affect fish health. A variety of heavy metals have been detected in water samples from the Delta Panhandle, including iron, nickel, copper, arsenic, selenium, and zinc, along with titanium, cadmium, lead, barium, and antimony [22, 51]. Contamination by pesticides and phthalates have also been reported in the Okavango [52–54]. Generally speaking, contaminant levels are low, but they likely fluctuate with changing flood conditions, such as decreases in pH that influence bound and free fractions of contaminants in the water column and sediments. In particular, iron levels in the environment are likely to contribute to iron accumulation in tissues. Likewise, contaminants like arsenic can induce inflammatory effects resembling those observed in this study, although at mg/L doses, which are much higher than the μg/L arsenic concentrations previously observed in the Delta [51, 55]. Taken together, we conclude that hypoxia is likely the main cause of the observed pathology in Okavango fishes, but we cannot rule out the synergistic effects of other co-stressors.

To date, population-level effects of the hypoxic stress have not been measured in Okavango fishes, but life spans of individual fish may be shortened. Perhaps more importantly, our findings identify seasonal hypoxia as a recurring natural stressor that may limit ecosystem resilience as human impacts rise. Given that fish health is already compromised by natural recurring hypoxia, they could be less able to overcome additional challenges from invasive species, upstream agriculture, contaminants, biodiversity losses, timber harvesting, sedimentation, frequent wildfires, overfishing, water diversion, dam construction, and climate change, all of which are progressing vividly in the Okavango [1]. Understanding the timing and impact of flood-induced hypoxia on Okavango fisheries can inform management decisions that promote sustainability of this exceptional and irreplaceable resource.

Additionally, an intact and ecologically functioning Okavango is essential to the wellbeing and economic security of the people of Botswana. In the form of tourism dollars, local natural resource use, and regional ecosystem services, the region contributes at least 7–8% of the country’s gross domestic product [56, 57]. This estimation is based on limited available economic data: in 2006 (the most recent year for which specific data are available), the Okavango Ramsar site contributed 2.6% of Botswana’s gross domestic product (GDP), in the form of tourism dollars, local natural resource use, and regional ecosystem services [56]. In 2019, tourism alone contributed 10.4% of Botswana’s GDP with about half coming from the Okavango Delta [57]. Taken together, these data suggest that total resources of the Delta currently provide at least 7–8% of Botswana’s GDP.

## Supporting information

S1 Table Sampling dates, GPS coordinates, approximate hypoxia timing, site water quality data, and fish demographics/morphometrics. (XLSX)

S2 Table Scoring criteria (characteristics and score range) to define categories of inflammation and cellular damage in the livers, spleens, and gonads of sampled Okavango fishes. (DOCX)

S1 Fig PowerPoint version of Fig 2 showing photographs of hepatic iron categories in higher resolution. (PPTX)

S2 Fig PowerPoint version of Fig 3 showing photographs of splenic iron categories in higher resolution. (PPTX)

S3 Fig PowerPoint version of Fig 4 showing photographs of gonadal iron categories in higher resolution. (PPTX)

S4 Fig PowerPoint version of Fig 7 showing photographs of hepatic inflammation in higher resolution. (PPTX)

S5 Fig PowerPoint version of Fig 9 showing photographs of splenic inflammation in higher resolution. (PPTX)
