# Supplementary material for: Low oxygen: A (tough) way of life for Okavango fishes
Source: PLoS One. 2020 Jul 30;15(7):e0235667. doi: 10.1371/journal.pone.0235667 (PMC7392303; doi:10.1371/journal.pone.0235667)
Supplement: S2 Table — (DOCX) [file pone.0235667.s002.docx]

**S2 Table. Scoring criteria (characteristics and score range) to define categories of inflammation and cellular damage in the livers, spleens, and gonads of sampled Okavango fishes.** (H&E = hematoxylin and eosin; MMAs = melanomacrophage aggregates; UGA = University of Georgia)

| **Characteristic** | **Score Range** | **Category** |
| --- | --- | --- |
| **Liver** | | |
| Proportion of acinar bundles encumbered with granulocytes in H&E stained liver sections. Examples shown in Fig 7. | 0 (none), 1 (sparse), 2 (< 50%), 3 (> 50%), or 4 (all) | Scores for each of the three characteristics were summed and aggregated into three categories.  **Low** (most healthy): total score 2-5 (n = 13). Low scoring fish tended to lack fibrosis but still exhibited granulocyte infiltration that was elevated above baseline levels observed in the UGA Aquaculture samples. Note that no fish received a score of 0 or 1.  **Medium**: total score 6-7 (n = 90); score due more to the abundance of granulocytes than ceroid/non-iron MMAs. All except four fish exhibited fibrosis.  **High** (most impaired): total score 8-9 (n = 118). |
| Abundance of diffuse brown ceroid/cellular debris and non-iron MMAs in liver sections stained with Perl’s Prussian blue. Examples shown in Fig 7H, 7K. | 0, 1, 2, 3 for lesions that were absent, low, medium, or high in density, respectively.  Low density lesions required the 40x objective to visualize, medium-density lesions were visible with the 10x objective, and high-density lesions were easily visible at the lowest magnification of 4x. |  |
| Fibrosis and/or thickened blood vessel or bile duct walls in the liver. Examples shown in Fig 7D, 7E, 7F. | 1 (present) or 0 (absent) with additional score of +1 for severe fibrosis, thickening, granulocyte infiltration, or evidence of necrosis. |  |
| **Spleen** | | |
| Spleen granulocytes (H&E stained). Examples shown in Fig 9B, 9C, 9F. | 1 (present) or 0 (absent) | Scores for each of the three characteristics were summed and aggregated into four categories.  **Low** (most healthy): total score 0-3 (n = 20). Low scoring fish tended to lack fibrosis and granulocyte infiltration, but still had ceroid and MMAs. A score of 0 applied to two UGA fish.  **Low-Medium**: total score = 4 (n = 19); Fish tended to have fibrosis, ceroid and MMAs, but lacked granulocytes.  **Medium**: total score = 5 (n = 63); Fish had all pathological characteristics, but not at the severe/widespread levels seen in the “high” category.  **High** (most impaired): total score 8-9 (n = 119). |
| Abundance of diffuse brown ceroid/cellular debris and non-iron MMAs in spleen sections stained with Perl’s Prussian blue. Example shown in Fig 9E. | 0, 1, 2, 3, or 4 using same framework as liver.  Because MMAs are common findings in normal fish spleens (see Fig 9A), the relative abundance scoring for spleen was shifted to the high end, compared with liver, which is why a “very high” score of 4 was included for spleens only. |  |
| Fibrosis and/or thickened blood vessel or bile duct walls in the spleen. Examples shown in Fig 9B-F. | 1 (present) or 0 (absent) with additional score of +1 for severe fibrosis, thickening, granulocyte infiltration, or evidence of necrosis. |  |
| **Gonad** | | |
| Abundance of diffuse brown ceroid/cellular debris and non-iron MMAs in gonad sections stained with Perl’s Prussian blue. | 1 (present) or 0 (absent) | No categories were defined because gonads were minimally or not affected by lesions associated with inflammation or cellular damage. |
| Granulocytes (H&E stained) | 1 (present) or 0 (absent) |  |
