# Supplementary material for: Low oxygen: A (tough) way of life for Okavango fishes
Source: PLoS One. 2020 Jul 30;15(7):e0235667. doi: 10.1371/journal.pone.0235667 (PMC7392303; doi:10.1371/journal.pone.0235667)
Supplement: S1 Fig — (PPTX) [file pone.0235667.s003.pptx]

## Slide 1
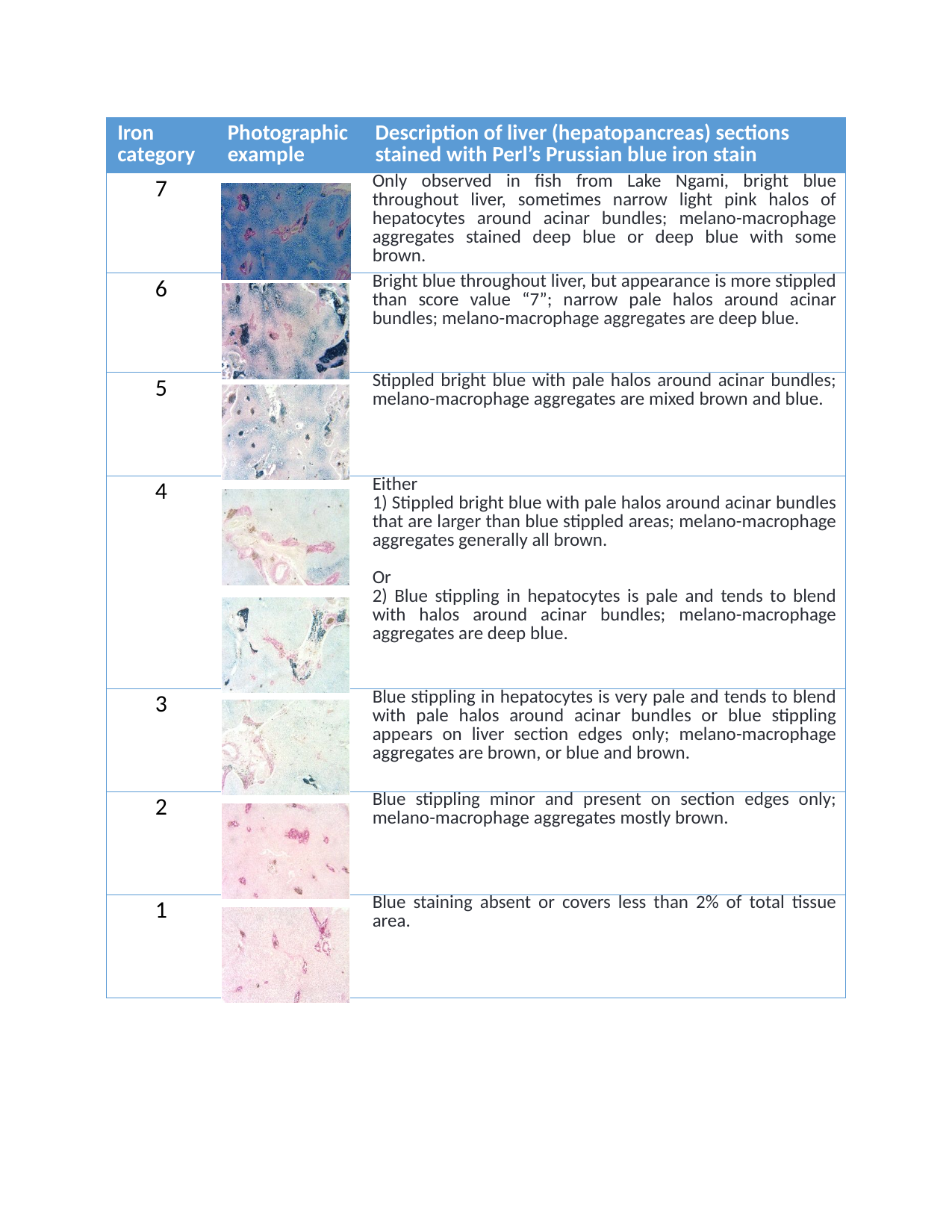

| Iron category | Photographic example | Description of liver (hepatopancreas) sections stained with Perl’s Prussian blue iron stain |
| --- | --- | --- |
| 7 | | Only observed in fish from Lake Ngami, bright blue throughout liver, sometimes narrow light pink halos of hepatocytes around acinar bundles; melano-macrophage aggregates stained deep blue or deep blue with some brown. |
| 6 | | Bright blue throughout liver, but appearance is more stippled than score value “7”; narrow pale halos around acinar bundles; melano-macrophage aggregates are deep blue. |
| 5 | | Stippled bright blue with pale halos around acinar bundles; melano-macrophage aggregates are mixed brown and blue. |
| 4 | | Either 1) Stippled bright blue with pale halos around acinar bundles that are larger than blue stippled areas; melano-macrophage aggregates generally all brown. Or 2) Blue stippling in hepatocytes is pale and tends to blend with halos around acinar bundles; melano-macrophage aggregates are deep blue. |
| 3 | | Blue stippling in hepatocytes is very pale and tends to blend with pale halos around acinar bundles or blue stippling appears on liver section edges only; melano-macrophage aggregates are brown, or blue and brown. |
| 2 | | Blue stippling minor and present on section edges only; melano-macrophage aggregates mostly brown. |
| 1 | | Blue staining absent or covers less than 2% of total tissue area. |
