# Supplementary material for: Low oxygen: A (tough) way of life for Okavango fishes
Source: PLoS One. 2020 Jul 30;15(7):e0235667. doi: 10.1371/journal.pone.0235667 (PMC7392303; doi:10.1371/journal.pone.0235667)
Supplement: S2 Fig — (PPTX) [file pone.0235667.s004.pptx]

## Slide 1
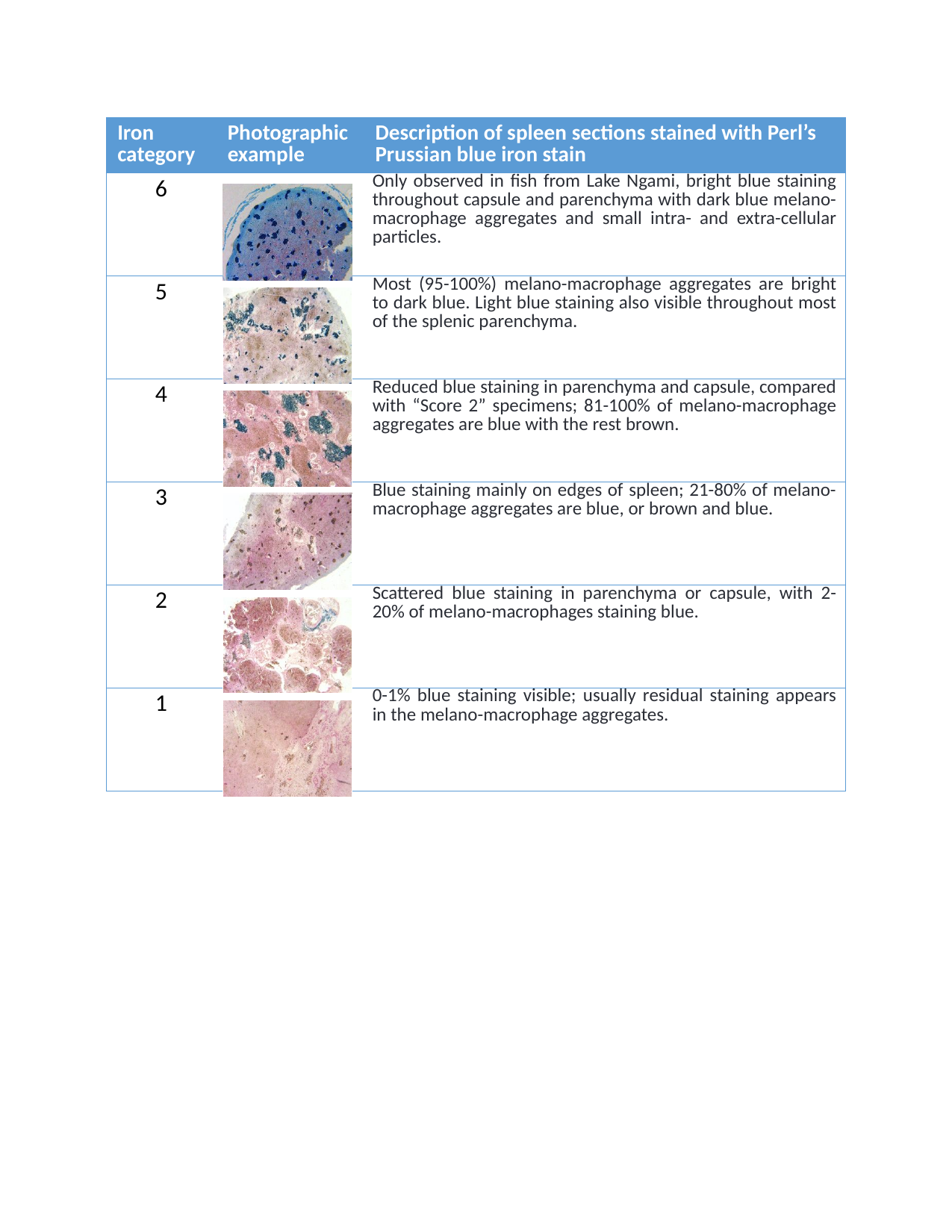

| Iron category | Photographic example | Description of spleen sections stained with Perl’s Prussian blue iron stain |
| --- | --- | --- |
| 6 | | Only observed in fish from Lake Ngami, bright blue staining throughout capsule and parenchyma with dark blue melano-macrophage aggregates and small intra- and extra-cellular particles. |
| 5 | | Most (95-100%) melano-macrophage aggregates are bright to dark blue. Light blue staining also visible throughout most of the splenic parenchyma. |
| 4 | | Reduced blue staining in parenchyma and capsule, compared with “Score 2” specimens; 81-100% of melano-macrophage aggregates are blue with the rest brown. |
| 3 | | Blue staining mainly on edges of spleen; 21-80% of melano-macrophage aggregates are blue, or brown and blue. |
| 2 | | Scattered blue staining in parenchyma or capsule, with 2-20% of melano-macrophages staining blue. |
| 1 | | 0-1% blue staining visible; usually residual staining appears in the melano-macrophage aggregates. |
