# Supplementary material for: Low oxygen: A (tough) way of life for Okavango fishes
Source: PLoS One. 2020 Jul 30;15(7):e0235667. doi: 10.1371/journal.pone.0235667 (PMC7392303; doi:10.1371/journal.pone.0235667)
Supplement: S3 Fig — (PPTX) [file pone.0235667.s005.pptx]

## Slide 1
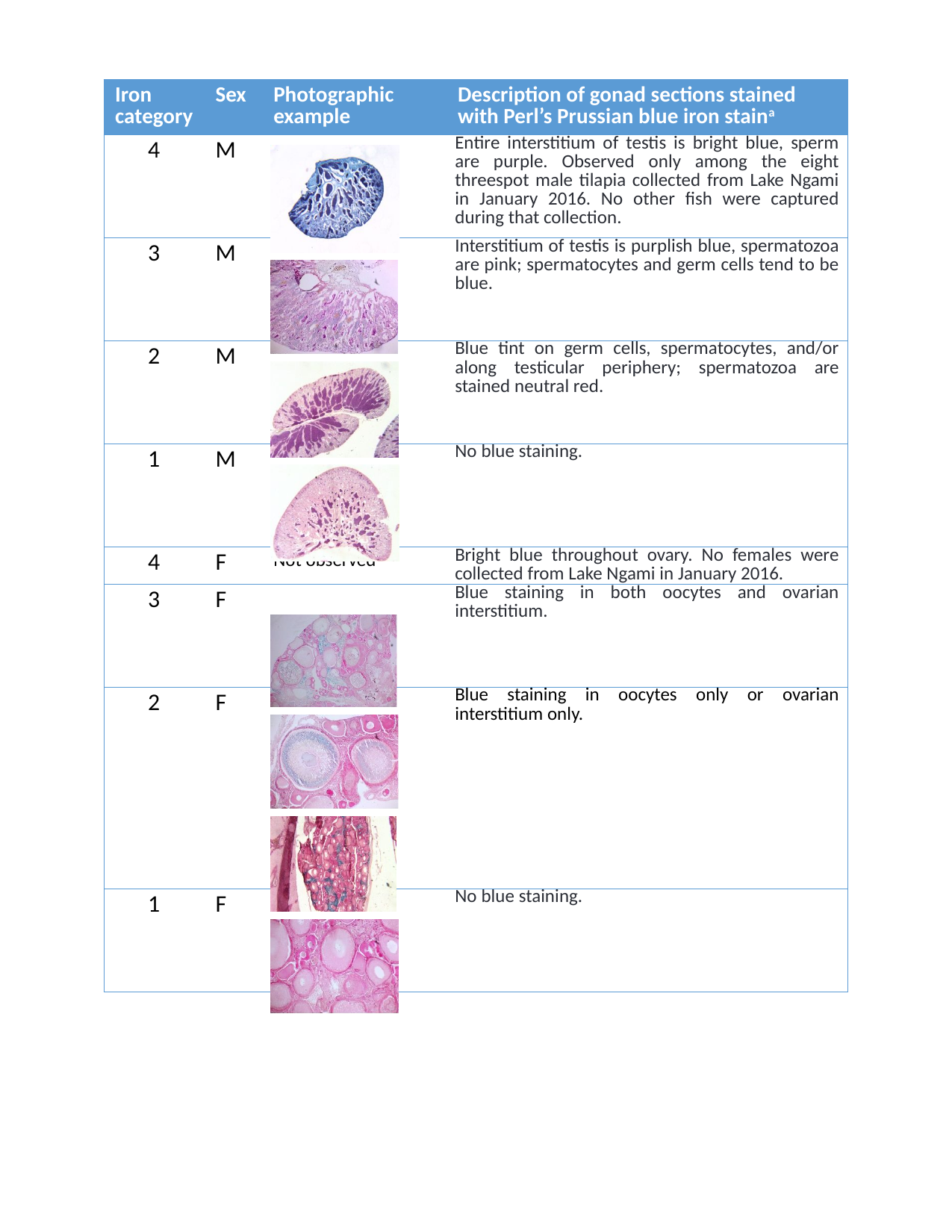

| Iron category | Sex | Photographic example | Description of gonad sections stained with Perl’s Prussian blue iron staina |
| --- | --- | --- | --- |
| 4 | M | | Entire interstitium of testis is bright blue, sperm are purple. Observed only among the eight threespot male tilapia collected from Lake Ngami in January 2016. No other fish were captured during that collection. |
| 3 | M | | Interstitium of testis is purplish blue, spermatozoa are pink; spermatocytes and germ cells tend to be blue. |
| 2 | M | | Blue tint on germ cells, spermatocytes, and/or along testicular periphery; spermatozoa are stained neutral red. |
| 1 | M | | No blue staining. |
| 4 | F | Not observed | Bright blue throughout ovary. No females were collected from Lake Ngami in January 2016. |
| 3 | F | | Blue staining in both oocytes and ovarian interstitium. |
| 2 | F | | Blue staining in oocytes only or ovarian interstitium only. |
| 1 | F | | No blue staining. |
