# Supplementary figures and images for: Low oxygen: A (tough) way of life for Okavango fishes
Source: PLoS One. 2020 Jul 30;15(7):e0235667. doi: 10.1371/journal.pone.0235667 (PMC7392303; doi:10.1371/journal.pone.0235667)

## Slide 1
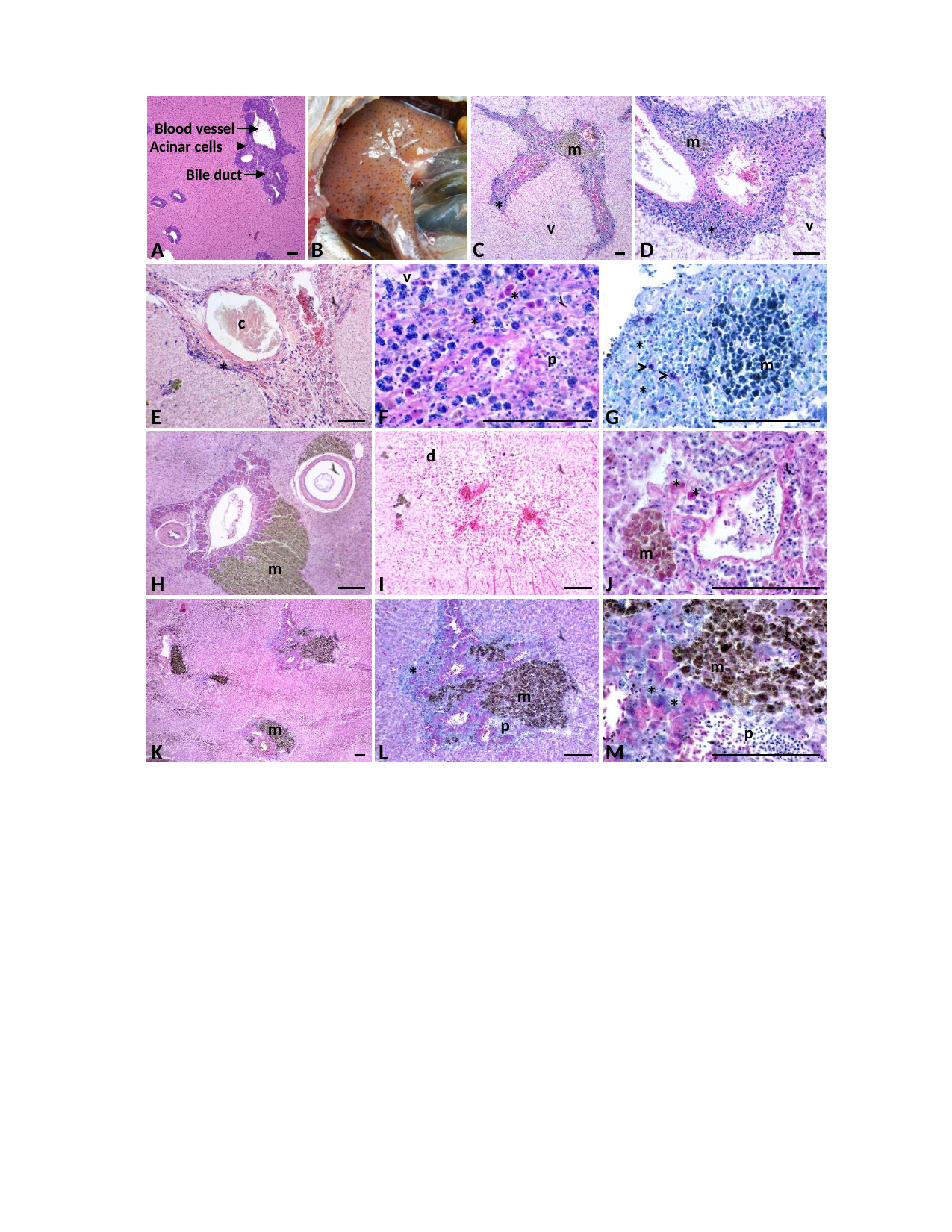

Blood vessel
m
Acinar cells
m
Bile duct
*
v
v
*
A
C
D
B
v
*
*
c
*
p
>
m
*
>
*
E
F
G
d
*
*
m
m
H
I
J
m
*
*
m
*
p
m
p
K
L
M

Supplement: S4 Fig — (PPTX) [file pone.0235667.s006.pptx]

## Slide 1
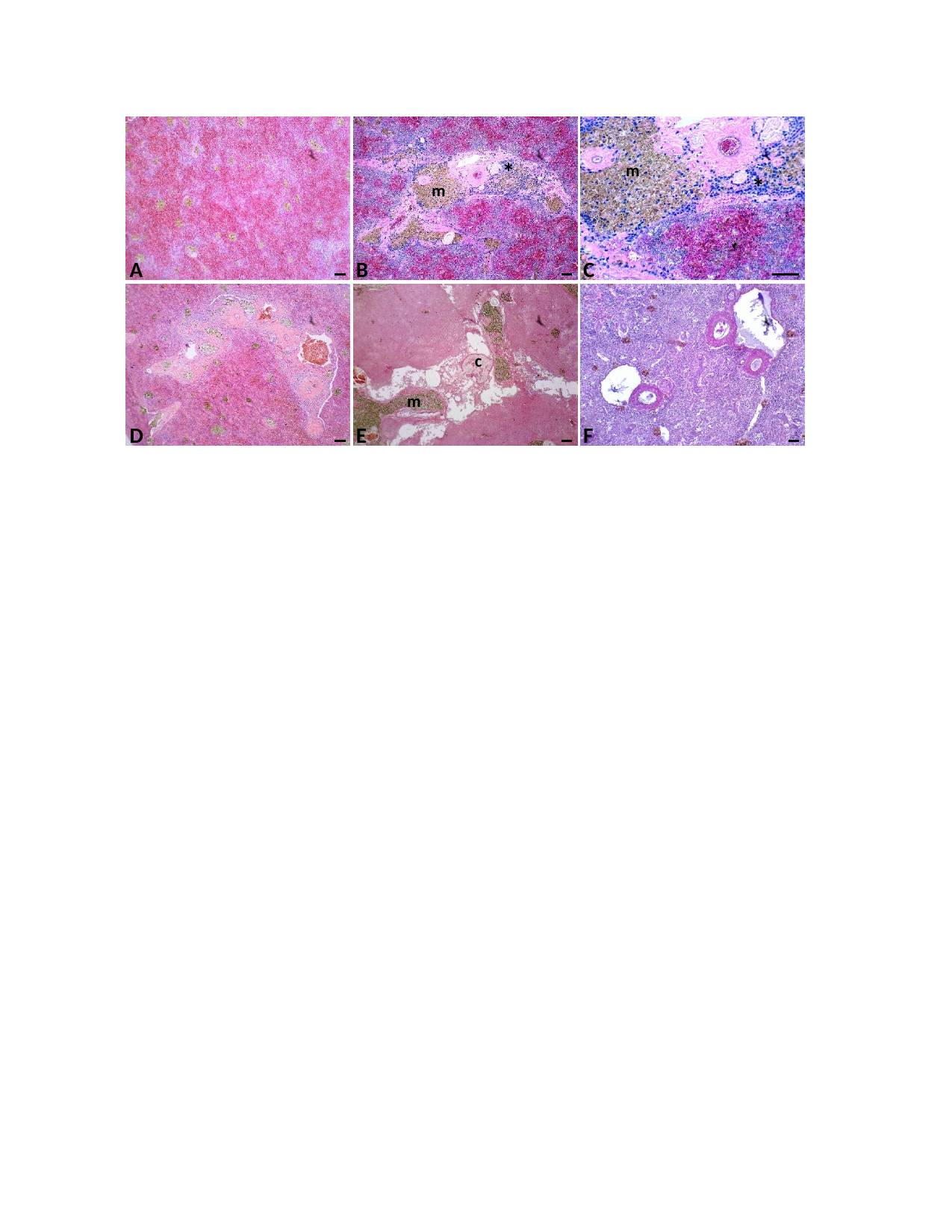

A
B
C
D
E
F
*
m
*
m
c
m

Supplement: S5 Fig — (PPTX) [file pone.0235667.s007.pptx]
